# Chemical Spill Encircling Using a Quadrotor and Autonomous Surface Vehicles: A Distributed Cooperative Approach

**DOI:** 10.3390/s22062178

**Published:** 2022-03-10

**Authors:** Marcelo Jacinto, Rita Cunha, António Pascoal

**Affiliations:** Laboratory of Robotics and Systems in Engineering and Science (LARSyS), Instituto Superior Técnico, University of Lisbon, 1049-001 Lisboa, Portugal; rita@isr.tecnico.ulisboa.pt (R.C.); antonio@isr.tecnico.ulisboa.pt (A.P.)

**Keywords:** quadrotor control, autonomous surface vehicle control, cooperative path following, online path planning, chemical spill boundary encircling

## Abstract

This article addresses the problem of formation control of a quadrotor and one (or more) marine vehicles operating at the surface of the water with the end goal of encircling the boundary of a chemical spill, enabling such vehicles to carry and release chemical dispersants used during ocean cleanup missions to break up oil molecules. Firstly, the mathematical models of the Medusa class of marine robots and quadrotor aircrafts are introduced, followed by the design of single vehicle motion controllers that allow these vehicles to follow a parameterised path individually using Lyapunov-based techniques. At a second stage, a distributed controller using event-triggered communications is introduced, enabling the vehicles to perform cooperative path following missions according to a pre-defined geometric formation. In the next step, a real-time path planning algorithm is developed that makes use of a camera sensor, installed on-board the quadrotor. This sensor enables the detection in the image of which pixels encode parts of a chemical spill boundary and use them to generate and update, in real time, a set of smooth B-spline-based paths for all the vehicles to follow cooperatively. The performance of the complete system is evaluated by resorting to 3-D simulation software, making it possible to visually simulate a chemical spill. Results from real water trials are also provided for parts of the system, where two Medusa vehicles are required to perform a static lawn-mowing path following mission cooperatively at the surface of the water.

## 1. Introduction

The problems of perimeter detection, boundary searching, and encircling have been widely researched topics with a variety of practical applications, ranging from the monitoring of wildfire spread in forests [1], to the control and encircling of oil spills [2] and harmful invasive algae blooms [3] at the surface of the ocean. In this paper, we will focus on the problem of chemical spill encircling.

The two main phenomena that contribute to the transportation and spread of hazardous chemicals over water, such as oil, are advection and diffusion. In the first, the chemical is transported due to the flow of water, while the second refers to the motion of the fluid caused by the existence of concentration gradients. One way of modelling the flow field of the incompressible fluid is by solving iteratively the convection–diffusion equations [4]. In the literature, many works address the problem of dynamic boundary tracking at the surface of the ocean by proposing control schemes which require (at least one) surface vessel to measure the concentration gradient of a hazardous contaminant. These measurements of the chemical plume are used by potential field controllers with the end goal of steering the robots to the boundary of the plume [5,6]. A completely different approach, adopted by Saldaña et al. [7], is to consider that a general environmental boundary can be approximated by a closed curve that is slowly varying over time and that can be described by a general parametric equation. In his research, the author proposes a model for the curve described spatially by a truncated Fourier series that changes its shape smoothly over time. To achieve this, it is assumed that a team of Autonomous Surface Vehicles (ASVs) are distributed equally around the chemical spill, and every vehicle is capable of taking local measurements of the boundary as it moves around it. These local measurements are then used to update the shape of the closed curve using recursive least squares. Although this is a very general solution to the problem, it can be argued that the use of a truncated Fourier series to represent a path for underactuated vehicles to follow is a rather poor choice of function, as the resulting curve can self-intersect and exhibit substantial oscillations. Moreover, it does not take into consideration the physical constraints imposed by the vehicles. In order to lift the limitations imposed by this method, more stable parametric curves could be considered, such as Bernstein polynomials or B-splines [8].

In recent years, there has been a massive development of and demand for Autonomous Underwater Vehicles (AUVs), due not only to their agility when it comes to the execution of scientific and comercial missions, but also to their low cost when compared to traditional ships, which require an on-board crew to be operated. Additionally, there has also been an exponential growth in demand for Unmanned Aerial Vehicles (UAVs), with a special emphasis on multirotor systems, which usually offer high-quality camera sensors at low market prices. Aerial vehicles can have a top-down view of the environment, making them the tool par excellence for surveillance and maintenance missions. On the other hand, AUVs and ASVs can be used to carry and release chemical dispersants used in cleanup missions to break oil molecules [9]. Together, these unmanned vehicles have huge potential to automate and reduce the cost of ocean cleanup operations.

**Figure 1:**
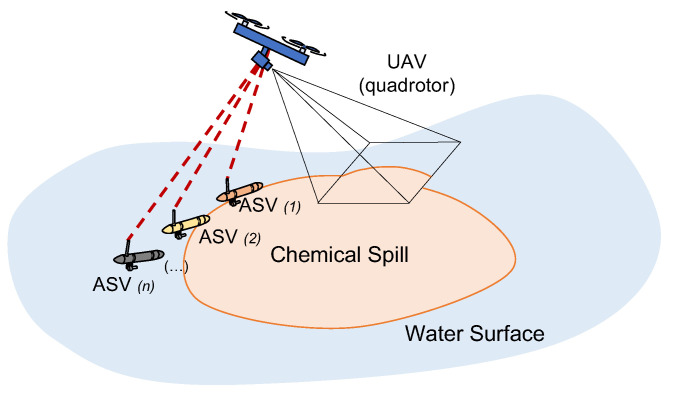
Cooperative path following along an environmental boundary.

In this paper, we address the problem of chemical spill encircling and focus on the development of a set of control and path planning tools that allow a team of robots constituted of a quadrotor (equipped with an onboard camera) and ASVs to detect and encircle the dynamical boundary of a chemical spill closely, as depicted in Figure 1. In our proposal, the quadrotor is responsible for detecting in real time the boundary of a chemical spill in the image stream produced by its onboard camera, and producing a path that itself and one or more ASVs are required to follow cooperatively. To achieve this, we start by proposing a set of single-vehicle motion control laws based on non-linear Lyapunov techniques that allow individual ASVs to follow a pre-defined parametric curve, based on previous works by Aguiar et al. [10,11,12]. These control techniques are then extended to the case of quadrotor vehicles. Borrowing from the work of N. Hung and F. Rego [13], a distributed controller using event-triggered communications is presented, allowing the vehicles to perform Cooperative Path Following (CPF) missions, according to a pre-defined geometric formation. Finally, a new real-time path planning framework that uses growing unclamped (and uniform) cubic B-splines is proposed, which fits a 2-D point cloud generated from the drone’s image stream.

A set of real experiments are performed with the Medusa class of marine vehicles [14] (property of ISR-DSOR) to access the real-life performance of the proposed path following and CPF algorithms. Additionally, the complete path planning solution is evaluated by resorting to the Gazebo 3-D simulator, PX4-SITL [15], and UUVSimulator [16], using a dynamic model of a Medusa vehicle and an Iris quadrotor equipped with a virtual RGB camera.

## 2. Preliminaries

### 2.1. Notation

The unit vector $e_{3}$ is defined as $e_{3}={\left[0,0,1\right]}^{T}$. For a vector $x\in R^{n}$, the symbol $x_{i}$ denotes the ith element of the vector. We shall use $∥x∥=\sqrt{x^{T}x}$ to denote the Euclidean norm of a vector. The notation K⪰0 is used to denote a matrix $K\in R^{n\times n}$ that is positive semi-definite. The symbol *I* is used to denote the identity matrix and 1 is a vector with all elements equal to one. The symbols ⌊x⌉,x∈R denote the *x* nearest integer, ⌊x⌋ denotes the floor of *x*, and ⌈x⌉ denotes the ceiling of *x*. The symbol R(.) is used to denote a rotation matrix with properties $R^{T}=R^{-1}$ and det(R)=1. The map $S\left(\cdot \right):R^{n}\to R^{n\times n},n=2,3$ yields a skew-symmetric matrix $S\left(x\right)y=x\times y,\forall x,y\in R^{n}$. When considering an estimator for an unknown variable *x*, we use the hat nomenclature $\hat{x}$ to denote its estimate and $\hat{x}$ when referring to the estimation error.

### 2.2. Graph Theory

A weighted digraph G=G(V,E,A) consists of a set of *N* vertices $V={\left[V_{1},...,V_{N}\right]}^{T}$, a set of directed edges E⊆V×V, and a weighted adjacency matrix $A=\left[a_{ij}\right]\in R^{N\times N}$, such that $a_{ij}>0$ if the edge that connects vertex *i* to *j* belongs to the graph, and 0 otherwise. The set of in-neighbours of a vertex *i* is given by $N_{i}^{in}=\left\{j\in V:\left(j,i\right)\in E\right\}$, and the set of out-neighbours by $N_{i}^{out}=\left\{j\in V:\left(i,j\right)\in E\right\}$. The in- and out-degree matrices $D^{in}$ and $D^{out}$ are a set of diagonal matrices defined by:

(1) $$D^{in/out}=diag\left(d_{i}^{in/out}\right),\;\mathrm{with}\;d_{i}^{in}=\sum_{j\in N_{i}^{in}}a_{ij}\;\mathrm{and}\;d_{i}^{out}=\sum_{j\in N_{i}^{out}}a_{ji}.$$

A graph G is undirected if communication links are unidirectional. If G is an undirected graph, then G is also balanced, i.e., $D^{in}=D^{out}:=D$, and its Laplacian matrix *L* is symmetric, positive semi-definite, and defined according to L:=(D−A). In these conditions, it is well known that *L* has a simple eigenvalue at zero associated with eigen vector 1, with the remaining eigen values being positive. Moreover, L1=0.

**Remark:** With the graph definition given above, we adopt the convention that an agent *i* can receive information from its neighbors in $N_{i}^{in}$ and send information to its neighbors in $N_{i}^{out}$.

### 2.3. Uniform B-Spline Curves

A 2-D B-spline curve of degree *k* + 1 in $R^{2}$ is a piecewise polynomial function formed by several components of degree *k*, defined as:

(2) $$C\left(\gamma \right)=\sum_{i=0}^{n}B_{i,k}\left(\gamma \right)P_{i},$$

where $P=\{P_{i}\in R^{2},i=0,...,n\}$ are a set of control points and $B_{i,k}\left(\gamma \right)$ are the B-spline basis functions. It follows from the Cox–De Boor’s recursive algorithm, according to L. Piegl and W. Tiller ([8], Chapter 2.2), that:

(3) $$B_{i,0}\left(\gamma \right)=\left\{\begin{array}{c} 1,\;\mathrm{if}\;\gamma_{i}\leq \gamma \leq \gamma_{i+1}\\ 0,\;\mathrm{otherwise} \end{array}\right.,$$

(4) $$B_{i,j}\left(\gamma \right)=\frac{\gamma -\gamma_{i}}{\gamma_{i+j}-\gamma_{i}}B_{i,j-1}\left(\gamma \right)+\frac{\gamma_{i+j+1}-\gamma }{\gamma_{i+j+1}-\gamma_{i+1}}B_{i+1,j-1}\left(\gamma \right),$$

where the index j=0,...,k and the values $\gamma_{i}$ belong to the *m*-dimensional knot vector $U={\left\{\gamma_{i}\right\}}_{i=0}^{m}$, with the number of knots related to the degree of the curve and the number of control points by m=k+1+n.

For the particular case of 2-D, uniform, non-clamped cubic B-splines with n−k+1 segments, each segment’s *x*- and *y*-coordinates of the parametric curve can be described according to the vectorial notation [17] as follows:

(5) $$C_{i}\left(\gamma \right):={\left[\begin{array}{cc} C_{i}^{x}\left(\gamma \right) & C_{i}^{y}\left(\gamma \right) \end{array}\right]}^{T},$$

with $C_{i}^{x}\left(\gamma \right)$ and $C_{i}^{y}\left(\gamma \right)$ computed according to:

(6) $$C_{i}^{x/y}\left(\gamma \right):=\underset{\left[\begin{array}{cccc} B_{i,3}\left(\gamma \right) & B_{i+1,3}\left(\gamma \right) & B_{i+2,3}\left(\gamma \right) & B_{i+3,3}\left(\gamma \right) \end{array}\right]}{\underset{︸}{\frac{1}{6}\left[\begin{array}{cccc} {\left(\gamma -i\right)}^{3} & {\left(\gamma -i\right)}^{2} & \left(\gamma -i\right) & 1 \end{array}\right]\left[\begin{array}{cccc} -1 & 3 & -3 & 1\\ 3 & -6 & 3 & 0\\ -3 & 0 & 3 & 0\\ 1 & 4 & 1 & 0 \end{array}\right]}}\left[\begin{array}{c} P_{i}^{x/y}\\ P_{i+1}^{x/y}\\ P_{i+2}^{x/y}\\ P_{i+3}^{x/y} \end{array}\right],$$

where γ∈[0,n−k+1] and i:=⌊γ⌋, such that γ−i∈[0,1] and each curve segment is only defined by four distinct control points. Defining a unidimensional vector with all control points $P={\left[P_{0}^{x},...,P_{n}^{x},P_{0}^{y},...,P_{n}^{y}\right]}^{T}\in R^{2(n+1)}$, where both *x*- and *y*-coordinates are concatenated, and a vector of distinct curve parameters $\gamma =\left[\gamma_{0},...,\gamma_{q}\right]\in R^{q+1}$ at which we wish to evaluate our curve, $C\left(\gamma \right)\in R^{2(q+1)}$ is given by:

(7) C(γ)=B(γ)·P,

where $B\left(\gamma \right)\in R^{2(q+1)\times 2(n+1)}$ is a diagonal by blocks matrix, and for each line of *B*, only four basis functions are different then zero and computed according to (6).

## 3. Vehicle Modelling

**Figure 2:**
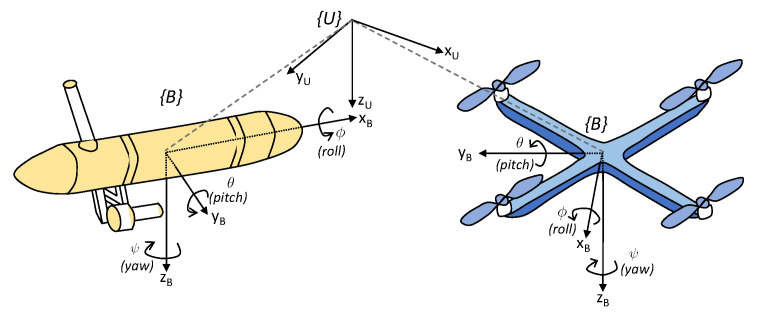
Adopted reference frames for a surface vehicle (**left**) and a quadrotor (**right**).

Let {U} denote an inertial reference frame and {B} a body-fixed reference frame attached to the geometric center of mass of each vehicle, according to Figure 2.

### 3.1. ASV Model

The ASV vehicle is modeled as a rigid body whose motion is restricted to a 2-D plane at the surface of the water, such that the roll and pitch angles are zero, i.e., ϕ=θ=0. Let the kinematic equations of the vehicle be given by:

(8) $$\underset{\dot{p}}{\underset{︸}{\left[\begin{array}{c} \dot{x}\\ \dot{y} \end{array}\right]}}=\underset{{}_{B}^{U}R\left(\psi \right)}{\underset{︸}{\left[\begin{array}{cc} cos(\psi ) & -sin(\psi )\\ sin(\psi ) & cos(\psi ) \end{array}\right]}}\underset{v}{\underset{︸}{\left[\begin{array}{c} u\\ v \end{array}\right]}}+\underset{v_{c}}{\underset{︸}{\left[\begin{array}{c} v_{cx}\\ v_{cy} \end{array}\right]}},$$

(9) $$\dot{\psi }=r,$$

where $p:={\left[x,y\right]}^{T}$ denotes the ASV position expressed in {U}, $v:={\left[u,v\right]}^{T}$ denotes the body-velocity vector, ${}_{B}^{U}R\left(\psi \right)\in R^{2\times 2}$ denotes the rotation matrix, and $v:={\left[v_{cx},v_{cy}\right]}^{T}$ denotes the ocean current, expressed in {U} and assumed to be constant, irrotational, and bounded. The ASV model is considered to be underactuated, with the input of the system being given by $u={\left[u,r\right]}^{T}\in R^{2}$.

### 3.2. Quadrotor Model

The kinematic equations that describe the motion of a rigid body in 3-D space can be described by a double integrator model, according to:

(10) $$\ddot{p}:=\underset{u}{\underset{︸}{ge_{3}-\frac{1}{m}{}_{B}^{U}R\left(\theta \right)Te_{3}}}+d,$$

where $p:={\left[x,y,z\right]}^{T}$ denotes the quadrotor’s position expressed in {U}, $\theta :={\left[\phi ,\theta ,\psi \right]}^{T}$ denotes the orientation vector of {B} expressed in {U}, and $u\in R^{3}$ can be regarded as the input of the system, comprising both the attitude of the vehicle and the total thrust *T*. The vector $d\in R^{3}$ represents unmeasured external disturbances, such as wind, acting on the vehicle, assumed to be constant and bounded such that $∥d∥\leq d_{max}$. The 3-D rotation matrix adopted for the quadrotor model follows the Z-Y-X convention, and is given by:

(11) $${}_{B}^{U}R(\theta )=R_{z}\left(\psi \right)R_{y}\left(\theta \right)R_{x}\left(\phi \right).$$

## 4. Path Following

**Figure 3:**
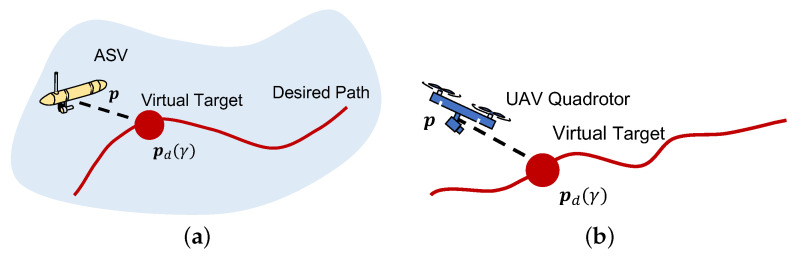
Path following schematic: (**a**) ASV path following. (**b**) Quadrotor path following.

The path following (PF) problem concerns the problem of making a vehicle move along a desired path $p_{d}\left(\gamma \right)$ parameterised by a variable γ (for example, the arc-length of the curve). The key idea is that each vehicle must approach a virtual target that moves along the path with a desired speed profile $v_{d}\left(\gamma \right)$, according to Figure 3. Since the end goal is to have more than one vehicle performing path following with a pre-defined inter-vehicle formation, this speed profile is given as the sum of another single-vehicle speed profile and an inter-vehicle coordination term, according to:

(12) $$v_{d}\left(\gamma \right):=v_{L}\left(\gamma \right)+v^{coord},\;\mathrm{with}\;\left|v_{L}\left(\gamma \right)\right|\leq v_{L}^{max},$$

where $v_{L}\left(\gamma \right)$ is a desired speed profile defined only as a function of the path, $v_{L}^{max}$ is a pre-defined speed upper-bound, and $v^{coord}$ is the speed coordination term that will be used in Section 5 to enable the CPF behaviour. It is important to notice that the desired speed profile $v_{L}\left(\gamma \right)$ should be the same for all the vehicles, enabling them to follow a given path at the same rate. On the other hand, the speed coordination term $v^{coord}$ will not be the same for all vehicles and will be used to adjust the progression speed of each individual robot based on how aligned they are with each other.

**Remark** **1.** *Speed profile $v_{d}\left(\gamma \right)$ might not correspond directly to an inertial speed, especially if the curve is not parameterised in terms of the arc-length. Nonetheless, a relation between the inertial speed and the desired speed profile is addressed in detail in Section 6.4.*

**Problem** **1.** *Given a generic vehicle (ASV or quadrotor), consider the geometric path $p_{d}\left(\gamma \right):\left[0,\infty \right]\to R^{2}/R^{3}$ for the ASV/quadrotor respectively, parameterised by a continuous variable γ∈R and $v_{d}\left(\gamma ,t\right)\in R$ a desired speed profile for a virtual target moving along the desired path. Furthermore, consider $p_{d}\left(\gamma \right)$ to be $C^{2}$ and have its first and second derivatives with respect to γ bounded. Assume the vehicle is equipped with inner-loop controllers allowing it to track a desired control reference $u_{d}\in R^{2}/R^{3}$, assumed to be bounded, by recruiting the appropriate forces and torques to apply to the vehicle. Design a feedback control law for the system input $u_{d}$ and virtual target $\ddot{\gamma }$ such that:*

- *the vehicle’s position converges to a tube around the desired position that can be made arbitrarily small, i.e., $∥p\left(t\right)-p_{d}\left(\gamma \right)∥$ converges to a neighbourhood of the origin;*
- *the speed of the virtual target moving along the path converges to the desired speed profile, i.e., $|\dot{\gamma }-v_{d}\left(\gamma ,t\right)|\to 0$ as t→∞.*

### 4.1. ASV Path Following

Following the approach proposed by Aguiar et al. [10,11,12], consider the global diffeomorphic coordinate transformation which expresses the position error defined in the body-frame of the vehicle {B} as:

(13) $$e_{p}\left(t\right):={}_{U}^{B}R(\psi )\left(p\left(t\right)-p_{d}\left(\gamma \right)\right),$$

and let the speed-tracking error be defined as:

(14) $$e_{\gamma }:=\dot{\gamma }-v_{d}\left(\gamma ,t\right).$$

With these definitions, the body-fixed position error dynamics are given by:

(15) $${\dot{e}}_{p}\left(t\right)={}_{U}^{B}\dot{R}\left(\psi \right)\left(p\left(t\right)-p_{d}\left(\gamma \right)\right)+{}_{U}^{B}R(\psi )\left(\dot{p}\left(t\right)-{\dot{p}}_{d}\left(\gamma \right)\right).$$

We recall that the derivative of a rotation matrix can be expressed as the product of a skew-symmetric matrix with the transposed rotation matrix, that is:

(16) $${}_{U}^{B}\dot{R}\left(\psi \right)=-S\left(r\right){}_{U}^{B}R\left(\psi \right).$$

Replacing (16) in (15) yields the position error dynamics expressed in the body-fixed frame as:

(17) $${\dot{e}}_{p}\left(t\right)=-S\left(r\right)\underset{e_{p}\left(t\right)}{\underset{︸}{{}_{U}^{B}R\left(\psi \right)\left(p\left(t\right)-p_{d}\left(\gamma \right)\right)}}+v+\underset{v_{c}}{\underset{︸}{{}_{U}^{B}R(\psi )v_{c}}}-{}_{U}^{B}R(\psi )\frac{\partial p_{d}\left(\gamma \right)}{\partial \gamma }\dot{\gamma }.$$

Since there is no direct control in the sway motion, the goal is to generate surge speed and heading rate control references. Therefore, we must make these references appear explicitly in the error expression. By introducing an offset $\delta ={\left[0,\delta \right]}^{T}\in R^{2}$ (with δ<0) in the standard position error, it is possible to re-write (17) as:

(18) $${\dot{e}}_{p}\left(t\right)=-S\left(r\right)\left(e_{p}-\delta \right)+\underset{\Delta }{\underset{︸}{\left[\begin{array}{cc} 1 & 0\\ 0 & -\delta \end{array}\right]}}\underset{u}{\underset{︸}{\left[\begin{array}{c} u\\ r \end{array}\right]}}+\left[\begin{array}{c} 0\\ v \end{array}\right]+v_{c}-{}_{U}^{B}R(\psi )\frac{\partial p_{d}\left(\gamma \right)}{\partial \gamma }\dot{\gamma }.$$

Consider that each ASV is equipped with a Doppler Velocity Logger (DVL) capable of providing the vehicle’s relative velocity with respect to the water v, expressed in {B}, and a Global Positioning System (GPS) unit which provides measurements of the position of the vehicle p, expressed in {U}. To estimate the ocean current, Pascoal et al. [18] and Sanches et al. [19] propose the use of a complementary filter. Consider the process model given by (8) and the candidate complementary filter model described by:

(19) $$F:=\left\{\begin{array}{c} \dot{\hat{p}}=k_{1}\left(p-\hat{p}\right)+{}_{B}^{U}R\left(\psi \right)v+{\hat{v}}_{c}\\ {\dot{\hat{v}}}_{c}=k_{2}\left(p-\hat{p}\right), \end{array}\right.$$

with $k_{1}$ and $k_{2}$ positive constants. The proposed complementary filter is asymptotically stable. For a formal stability analysis of this complementary filter, refer to Pascoal et al. [18].

At this point, it is important to notice that the current velocity $v_{c}$ and the requested input $u_{d}$ that are to be applied to a vehicle’s kinematic model cannot be estimated and tracked, respectively, with infinite precision. For this reason, we define the current estimation error and the inner-loop tracking error given by:

(20) $$\begin{array}{cc} {\tilde{v}}_{c} & :=v_{c}-{\hat{v}}_{c},\\ \tilde{u} & :=u-u_{d}. \end{array}$$

Consider the Proposition 1 introduced below, in which a solution to Problem 1, applied to an ASV, is provided along with convergence guarantees in the presence of bounded estimation and tracking errors.

**Proposition** **1.** *Consider the system described by the kinematics in (8), with the outer-loop control laws given by:*

(21) $$u_{d}:=\Delta^{-1}\left(-K_{p}\sigma \left(e_{p}-\delta \right)-\left[\begin{array}{c} 0\\ v \end{array}\right]-{\hat{v}}_{c}+{}_{U}^{B}R\left(\psi \right)\frac{\partial p_{d}\left(\gamma \right)}{\partial \gamma }v_{d}\left(\gamma ,t\right)\right),$$

(22) $$\ddot{\gamma }:=-k_{\gamma }e_{\gamma }+{\dot{v}}_{d}\left(\gamma ,t\right)+{\left(e_{p}-\delta \right)}^{T}{}_{U}^{B}R\left(\psi \right)\frac{\partial p_{d}\left(\gamma \right)}{\partial \gamma },$$

*where $K_{p}⪰0$, $k_{\gamma }>0$, and $\sigma \left(e_{p}\right)=tanh\left(∥e_{p}∥\right)\frac{e_{p}}{∥e_{p}∥}$ is a saturation function. The closed-loop system is input-to-state stable (ISS) with respect to $\Delta \tilde{u}+{\tilde{v}}_{c}$, and the proposed control law solves Problem 1 for the ASV vehicle.*

**Proof.**  Appendix A.    □

### 4.2. Quadrotor Path Following

Given that the quadrotor system is modelled by a double integrator in the inertial frame {U}, as stated in (10), consider the position and velocity errors defined in {U} as:

(23) $$e_{p}:=p\left(t\right)-p_{d}\left(\gamma \right),$$

(24) $$e_{v}:=\dot{p}-\frac{\partial p_{d}}{\partial \gamma }v_{d}\left(\gamma ,t\right),$$

and a virtual target speed tracking error defined by (14). Consider also a new auxiliary error z, defined as:

(25) $$z:=e_{v}+K_{1}e_{p},$$

where $K_{1}⪰0$ is a gain matrix. The position and velocity error dynamics can be written as:

(26) $${\dot{e}}_{p}=\dot{p}-\frac{\partial p_{d}}{\partial \gamma }\dot{\gamma },$$

(27) $${\dot{e}}_{v}=\ddot{p}-\frac{d}{dt}\left(\frac{\partial p_{d}}{\partial \gamma }v_{d}\left(\gamma ,t\right)\right).$$

Furthermore, consider the time derivative introduced in (27), the desired virtual target speed function (12), and the virtual target speed tracking error function (14). Then, the time derivative term introduced in (27) can be expanded as:

(28) $$\begin{array}{cc} \frac{d}{dt}\left(\frac{\partial p_{d}}{\partial \gamma }v_{d}\left(\gamma ,t\right)\right) & =\left[\underset{h(\gamma )}{\underset{︸}{\frac{\partial^{2}p_{d}}{\partial \gamma^{2}}v_{d}\left(\gamma ,t\right)+\frac{\partial p_{d}}{\partial \gamma }\frac{\partial v_{L}\left(\gamma \right)}{\partial \gamma }}}\right]\left(e_{\gamma }+v_{d}\left(\gamma ,t\right)\right)+\frac{\partial p_{d}}{\partial \gamma }{\dot{v}}^{coord}\left(t\right). \end{array}$$

Replacing (10) and (28) in (27) yields:

(29) $${\dot{e}}_{v}=u+d-h\left(\gamma \right)\left(e_{\gamma }+v_{d}\left(\gamma ,t\right)\right)-\frac{\partial p_{d}}{\partial \gamma }{\dot{v}}^{coord}\left(t\right).$$

Unlike the case of the ASVs where current estimates are given by a complementary filter, in the case of a quadrotor, a different direction is taken towards estimating disturbances such as wind. According to Xie and Cabecinhas et al. [20,21], straightforward implementations of estimators can lead to windup and result in unbounded growth of an external disturbance estimate. To avoid such problems, Xie and Cabecinhas propose the use of a sufficiently smooth projection operator in the estimator design. Consider the disturbance observer given by:

(30) $$\dot{\hat{d}}:=K_{d}\mathrm{Proj}\left(z,\hat{d}\right)=z-\frac{\eta_{1}\eta_{2}}{2{\left(\beta^{2}+2\beta d_{max}\right)}^{n+1}d_{max}^{2}}\hat{d},$$

where $K_{d}$ denotes a diagonal gain matrix and:

(31) $$\eta_{1}=\left\{\begin{array}{c} {\left({\hat{d}}^{T}\hat{d}-d_{max}^{2}\right)}^{n+1},\;\mathrm{if}\;\left({\hat{d}}^{T}\hat{d}-d_{max}^{2}\right)>0\\ 0,\;\mathrm{otherwise}, \end{array}\right.$$

and:

(32) $$\eta_{2}={\hat{d}}^{T}z+\sqrt{{\left({\hat{d}}^{T}z\right)}^{2}+\varsigma^{2}},$$

where ς,β>0 are arbitrary constants. This projection operator, first proposed in Cai et al. [22], enjoys the useful properties:

(33) $${\tilde{d}}^{T}Proj\left(z,\hat{d}\right)\geq {\tilde{d}}^{T}z,$$

and:

(34) $$∥\hat{d}∥\leq d_{max}+\beta ,\forall t\geq 0.$$

Once again, consider the inner-loop tracking error and disturbance estimation error given by:

(35) $$\tilde{u}:=u-u_{d},$$

(36) $$\tilde{d}:=d-\hat{d}.$$

**Proposition** **2.** *Consider the system described by (10), the disturbance estimator dynamics given by (30), and the inner-loop tracking error given by (35). Furthermore, consider the control law given by:*

(37) $$u_{d}:=-\hat{d}+h\left(\gamma \right)v_{d}\left(\gamma ,t\right)+\frac{\partial p_{d}}{\partial \gamma }{\dot{v}}^{coord}\left(t\right)-e_{v}K_{v}-e_{p}K_{p},$$

(38) $$\ddot{\gamma }:=-k_{\gamma }e_{\gamma }+{\dot{v}}_{d}\left(\gamma ,t\right)+e_{p}^{T}\frac{\partial p_{d}}{\partial \gamma }+z^{T}\left(h\left(\gamma \right)+K_{1}\frac{\partial p_{d}}{\partial \gamma }\right),$$

*where $K_{p},K_{v}⪰0$, and $k_{\gamma }$ is a positive gain. For sufficiently small initial position and velocity errors ($e_{p}$, $e_{v}$), and a sufficiently large separation between the time-scales of the inner and outer loop systems, it can be guaranteed that the system error converges to a neighbourhood of zero. The proposed control law solves Problem 1 for the quadrotor vehicle.*

**Remark** **2.** *In-depth and quantitative overall stability analysis can be conducted for the inner–outer loop control system, but this will be dependent directly on the type of inner loop adopted. This results from the fact that the desired accelerations $u_{d}$ must be decoupled in a set of desired thrust and attitude for the quadrotor to track. Given that this analysis is out of the scope of this work, we assume that the quadrotor is equipped with a generic inner loop that is capable of keeping the tracking error $\tilde{u}$ small and bounded.*

**Proof.**  Appendix B.    □

## 5. Cooperative Path Following

In this section, the problem of CPF is addressed. The end goal is to have an algorithm that allows one quadrotor and multiple ASVs to perform a path following mission cooperatively, using a distributed architecture. The vehicles are required to execute their mission according to a fixed geometric configuration. To cope with limitations imposed by real environments where inter-vehicle communications are discrete, an Event-Triggered Communications (ETC) mechanism is adopted, based on previous work developed by A. Aguiar and A. Pascoal [23] and N. Hung and F. Rego [13].

### Synchronisation Problem with Event-Triggered Communications

Consider a group of $N\in R^{+}\backslash \left\{1\right\}$ autonomous vehicles/agents in a network that can be described mathematically by a digraph G(V,E,A), consisting of *N* vertices, a set of directed edges E⊆V×V, where the edge $\varepsilon_{ij}$ represents the flow of information from agent *i* to agent *j*, and a weighted adjacency matrix $A=\left[a_{ij}\right]\in R^{N\times N}$. Furthermore, each vehicle *i* is able to receive information from its neighbours in $N_{i}^{in}$ and send information to its neighbours in $N_{i}^{out}$, i.e., G is undirected. Moreover, consider that the communication topology of the vehicles is fixed; hence, the Laplacian *L* associated to G is constant. Let the state vector of the system be composed by the path parameter of each individual vehicle $\gamma ={\left[\gamma_{1},...,\gamma_{N}\right]}^{T}$. In addition, each vehicle is equipped with the PF controllers proposed in Section 4, and has an assigned path to follow, appropriately parameterised in order to ensure that a given desired formation between the vehicles is met. The CPF problem consists in designing a distributed control scheme that adjusts the speed of the vehicles such that all path parameters γ reach a consensus. Consider the problem formulation below.

**Problem** **2.** *For each agent i, with i=1,...,N, derive a consensus protocol for the speed correction term $v^{\mathrm{coord}}={\left[v_{1}^{coord},...,v_{N}^{coord}\right]}^{T}$, such that $\lim_{t\to \infty }\left|\gamma_{i}-\gamma_{j}\right|=0$, $\forall j\in N_{i}^{in}$, and the formation of vehicles achieves the desired speed assignment $v_{L}\left(\gamma \right)={\left[v_{L1},...,v_{LN}\right]}^{T}$ as t→∞.*

Note that, according to the previously developed (PF) controllers, for each vehicle *i*, $|{\dot{\gamma }}_{i}-v_{d}\left(\gamma ,t\right)|=0$ is only guaranteed as t→∞, as the controlled variable is ${\ddot{\gamma }}_{i}$ and not ${\dot{\gamma }}_{i}$. Having this fact in mind, and assuming that the vehicles have already converged to their desired paths, i.e., $e_{p}\approx 0$ (and $e_{v}\approx 0$ in the case of the quadrotor), then the following simplifying assumption can be made:

**Assumption** **1.** *The speed progression of all the virtual targets along the desired path is always assumed to be modelled by a single integrator system, which can be expressed in vectorial form as:*

(39) $$\dot{\gamma }=v_{d}\left(\gamma ,t\right)=v_{L}\left(\gamma \right)+v^{\mathrm{coord}}.$$

Let the synchronisation error vector be defined as $\varepsilon ={\left[\varepsilon_{1},...,\varepsilon_{N}\right]}^{T}$ where, for each *i*:

(40) $$\varepsilon_{i}:=\sum_{j\in N_{i}^{in}}a_{ij}\left(\gamma_{i}-\gamma_{j}\right),$$

with $a_{ij}$ elements of the weighted adjacency matrix that describes the vehicle network. This error can also be expressed in vectorial form as:

(41) ε:=Lγ,

where $\varepsilon_{i}$ denotes the coordination error between vehicle *i* and its neighbours. With the above notation, the coordination error dynamics of the multi-vehicle system are given by:

(42) $$\dot{\varepsilon }:=L\dot{\gamma }.$$

In the work of N. Hung and F. Rego [13], the authors propose a scheme where each agent *i* has a set of estimators ${\hat{\gamma }}_{j},j\in N_{i}^{in}$ for the true state of each in-neighbour virtual target $\gamma_{j}$. In addition, each agent *i* has an estimator for its own state ${\hat{\gamma }}_{i}$, which is reset whenever vehicle *i* broadcasts its true state $\gamma_{i}$. The other estimators are reset whenever agent *i* receives the true state from its in-neighbours $j\in N_{i}^{in}$. In this work, a time-dependent broadcast condition is adopted.

**Proposition** **3.** *Consider the distributed control law given by:*

(43) $$v_{i}^{coord}:=-k_{\varepsilon }\sum_{j\in N_{i}^{in}}a_{ij}\left(\gamma_{i}-{\hat{\gamma }}_{j}\right),$$

*where $k_{\varepsilon }>0$ and ${\hat{\gamma }}_{j}$ is vehicle i’s estimate of vehicle j’s real virtual target value. Consider also that the bank of estimators that each vehicle i is running is described by the dynamics equation:*

(44) $${\dot{\hat{\gamma }}}_{i}:=v_{L}\left({\hat{\gamma }}_{i}\right).$$

*At any time instant t, under negligible transmission delays, the vehicle j’s self-state estimate ${\hat{\gamma }}_{j}$ is equal to vehicle i’s estimate of ${\hat{\gamma }}_{j}$, which allows us to express the estimator dynamics using vectorial notation as:*

(45) $$\dot{\hat{\gamma }}:=v_{L}\left(\hat{\gamma }\right),$$

*where $\hat{\gamma }={\left[{\hat{\gamma }}_{1},...,{\hat{\gamma }}_{N}\right]}^{T}$ is the self-estimate of the virtual target of each vehicle. Let $\tilde{\gamma }={\left[{\tilde{\gamma }}_{1},...,{\tilde{\gamma }}_{N}\right]}^{T}$ denote the local estimation errors of each vehicle, such that $\tilde{\gamma }=\gamma -\hat{\gamma }$. Then, $v^{\mathrm{coord}}$ can also be given in vectorial notation, according to:*

(46) $$v^{\mathrm{coord}}:=-k_{\varepsilon }\left[D\gamma -A\hat{\gamma }\right]=-k_{\varepsilon }\left(\varepsilon +A\tilde{\gamma }\right),$$

*where D is a diagonal matrix and A the graph adjacency matrix. Consider also a triggering function used to define when to broadcast the along-path position of the virtual target of each vehicle, defined as:*

(47) $$\left\{\begin{array}{c} \delta_{i}\left(t\right):=\left|{\tilde{\gamma }}_{i}\left(t\right)\right|-g_{i}\left(t\right)\\ {\tilde{\gamma }}_{i}\left(t\right)={\hat{\gamma }}_{i}\left(t\right)-\gamma_{i}\left(t\right), \end{array}\right.$$

*where ${\tilde{\gamma }}_{i}\left(t\right)$ is the local estimation error of agent i and $g_{i}\left(t\right)$ is a time-dependent threshold function, such that if the estimation error exceeds this threshold, i.e., $\delta_{i}\left(t\right)\geq 0$, vehicle i broadcasts its state to the out-neighbours $N_{i}^{out}$ and resets its local estimator. Furthermore, consider $g_{i}\left(t\right)$ to belong to a class of non-negative functions, given by:*

(48) $$g_{i}\left(t\right)=c_{i}+b_{i}e^{-\alpha_{i}t},$$

*with $c_{i}$, $b_{i}$ and $\alpha_{i}$ being positive constants and $g\left(t\right)={\left[g_{1},...,g_{N}\right]}^{T}$ being the collection of functions $g_{i}$ for each individual vehicle i. Consider also that $v_{L}\left(\gamma \right)=v_{L}1+{\tilde{v}}_{L}$, where ${\tilde{v}}_{L}$ is a bounded and arbitrarily small term that accounts for a transient period in which the vehicles are on different sections of the path, with slightly different desired speed profiles. Then, under Assumption 1, the system is ISS with respect to the error vector ε and the inputs $\tilde{\gamma }$ and ${\tilde{v}}_{L}$.*

**Proof.**  Appendix C.    □

The proposed control scheme used for achieving CPF using ETC is summarised in Algorithm 1.
**Algorithm 1** Event-Triggered Communication for vehicle *i* (adapted from [24]).1:At every time instant *t*, each vehicle *i* follows the procedure:2:**procedure **Coordination and Communication3:    **if** $\delta_{i}\left(t\right)\geq 0$ where $\delta_{i}$ is computed using (47) and (48) **then**4:        Broadcast $\gamma_{i}\left(t\right)$;5:        Reset the estimator ${\hat{\gamma }}_{i}$;6:    **if** Receive a new message from agent $j\in N_{i}^{in}$ **then**7:        Reset ${\hat{\gamma }}_{j}\left(t\right)$;8:    Run the estimators according to (44);9:    Update the first order control protocol $u_{i}$ using (43).

Given the general distributed control scheme, we now elaborate and address a specific formation, in the context of this work, in the sections that follow.

## 6. Path Planning

This section addresses the problem of generating a set of smooth and planar reference paths for each individual vehicle to follow, with the end goal of encircling the boundary of a chemical spill. In order to make the vehicles follow the dynamic boundary according to a pre-defined formation (such as a triangle) multiple paths should be generated from one reference path that encodes the boundary. Borrowing from the work of Saldaña et al. [7], we start by presenting a rigorous mathematical definition of a dynamic boundary below.

**Figure 4:**
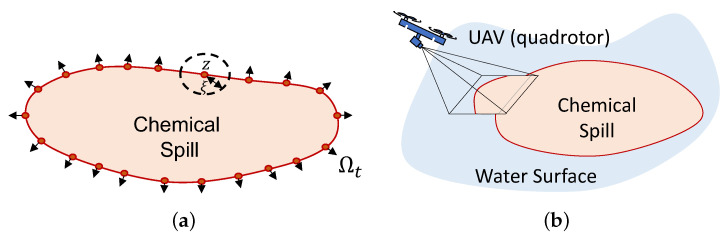
Dynamic Boundary schematic: (**a**) Boundary formal definition. (**b**) Drone’s field of view.

**Definition** **1.** *A dynamic boundary is a set of planar points $\Omega_{t}$, such that $\forall z\in \Omega_{t}$, and for any ξ>0, the open disk centered at point z with radius ξ contains points of $\Omega_{t}$ and its complement set $\Omega_{t}^{C}$. Moreover, the dynamic boundary can be approximated by a parametric closed curve (Jordan curve) $C\left(\gamma ,t\right):\left[0,\infty \right]\times \left[0,\infty \right]\to R^{2}$, mapped by a parameter $\gamma \in R_{0}^{+}$ and time $t\in R_{0}^{+}$. The curve is continuous with no self-intersecting points, and changes smoothly with respect to both time t and parameter γ, as depicted in Figure 4a.*

Since the chemical spill boundary is assumed to be dynamic, a path planning problem can be formulated in which a quadrotor is actively re-planning the path that the ASVs should follow at the water surface, as the group of vehicles moves along it and more up-to-date data is acquired by the quadrotor’s vision system. Consider, therefore, Problem 3.

**Problem** **3.** *Consider a quadrotor flying over a body of water at a pre-defined fixed altitude, equipped with a camera sensor pointing downwards with a fixed pitch angle relative to the vehicle’s body reference frame {B}. Consider also that the vehicle is capable of detecting the boundary of a chemical spill in the 2-D image provided by the camera sensor. Furthermore, one or more ASVs at the surface of the water are required to follow a path dictated by the quadrotor, according to a pre-defined vehicle formation. As the quadrotor detects the dynamic boundary in the image:*

1 *use the data provided by its navigation system to convert the pixels to a 2-D point cloud expressed in the inertial frame {U};*
2 *remove outliers and perform pre-processing on the 2-D point cloud;*
3 *generate a smooth and planar reference path by formulating an online optimisation problem that fits the data with open uniform B-splines;*
4 *send the updated path to the vehicle network;*
5 *make each vehicle generate an unique path for itself, capturing the pre-defined vehicle formation;*
6 *repeat the process.*

In order to solve Problem 3, a few simplifying assumptions are made:

**Assumption** **2.** *The dynamic boundary is located at the ocean’s surface, assumed to be a 2-D plane at $Z_{U}=0$ in the inertial frame of reference {U}.*

**Assumption** **3.** *The quadrotor has a navigation system that can track the vehicle’s pose with good accuracy.*

**Assumption** **4.** *The quadrotor has a limited field of view of the environment, i.e, the camera sensor might not be able to capture the entire chemical spill boundary, but rather sections of it, according to Figure 4b.*

**Assumption** **5.** *The detection of the pixels that encode the boundary in the image frame is a sub-system that is assumed to be already available, such as the one proposed in [25].*

### 6.1. Planar Point Cloud Generation

**Figure 5:**
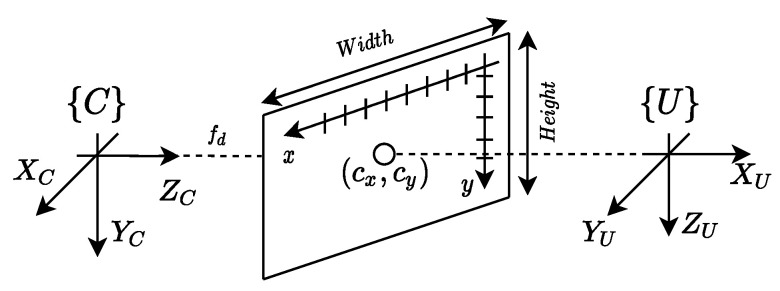
Camera model and reference frames.

The camera model adopted is characterised by: (i) a set of extrinsic parameters, which model the conversion between coordinates expressed in the world/inertial reference frame {U} and the camera reference frame {C}; (ii) intrinsic parameters which describe how a set of points in {C} are represented in the image frame, according to Figure 5.

The intrinsic parameters consist of the focal distance $f_{d}$, the scale factors ($s_{x}$, $s_{y}$) in the *X*- and *Y*-axis, respectively, and ($c_{x}$, $c_{y}$), which corresponds to the offset of the focal point in the image plane. These parameters can be obtained a priori by resorting to a camera calibration process, described in detail in [26]. Combining the matrices of intrinsic parameters *K*, also known as the full-rank calibration matrix, and the matrix of external parameters ${}_{U}^{C}\left[R|T\right]$, and expressing the inertial frame coordinates as homogeneous coordinates, the transformation between inertial frame and camera plane is described by:

(49) $$\lambda \left[\begin{array}{c} x\\ y\\ 1 \end{array}\right]=\underset{K}{\underset{︸}{\left[\begin{array}{ccc} f_{d}s_{x} & 0 & c_{x}\\ 0 & f_{d}s_{y} & c_{y}\\ 0 & 0 & 1 \end{array}\right]\left[\begin{array}{cccc} 1 & 0 & 0 & 0\\ 0 & 1 & 0 & 0\\ 0 & 0 & 1 & 0 \end{array}\right]}}\underset{{}_{U}^{C}\left[R|T\right]}{\underset{︸}{\left[\begin{array}{cc} {}_{U}^{C}R & {}_{U}^{C}T\\ 0_{1\times 3} & 1 \end{array}\right]}}\left[\begin{array}{c} X_{U}\\ Y_{U}\\ Z_{U}\\ 1 \end{array}\right],$$

where *x* and *y* denote the coordinates in the image frame and λ is a scale factor. It is important to mention that ${}_{U}^{C}\left[R|T\right]$ results from a series of successive rigid-body transformations (rotations and translations) given by:

(50) $${}_{U}^{C}\left[R|T\right]={}_{B}^{C}\left[R|T\right]{}_{U}^{B}\left[R|T\right],$$

where ${}_{U}^{B}\left[R|T\right]$ denotes the conversion of coordinates expressed in the inertial frame {U} to the quadrotor’s body frame {B}, provided by its navigation system, and ${}_{B}^{C}\left[R|T\right]$ is a matrix known a priori, as the camera attached to the vehicle is assumed to be fixed. The intrinsic and extrinsic parameters can be aggregated in a matrix Ω according to:

(51) $$\Omega =K\cdot {}_{U}^{C}\left[R|T\right].$$

In order to convert a given set of pixels (x,y) that encode the chemical spill boundary in the image frame to a point cloud expressed in the inertial frame, depth information about the scene is required. Taking into consideration Assumption 2, all the points in the inertial frame will lie on the plane described by $Z_{U}=0$, which solves the depth requirement. Moreover, from Assumption 3, it can be concluded that the linear system of Equation (49) is well defined and can be inverted such that for each pixel representing the boundary of the chemical spill, $X_{U}$ and $Y_{U}$ are extracted from:

(52) $$\frac{1}{\lambda }\left[\begin{array}{c} X_{U}\\ Y_{U}\\ 1 \end{array}\right]={\left[\begin{array}{ccc} \Omega_{1} & \Omega_{2} & \Omega_{4}\\ \Omega_{5} & \Omega_{6} & \Omega_{8}\\ \Omega_{9} & \Omega_{10} & \Omega_{12} \end{array}\right]}^{-1}\left[\begin{array}{c} x\\ y\\ 1 \end{array}\right].$$

**Remark** **3.** *This methodology relies heavily on the assumption that the quadrotor has a good navigation system, since small estimation errors in the altitude of the vehicle can lead to errors of several meters in the generated point cloud.*

### 6.2. Pre-Processing the Planar Point Cloud

**Figure 6:**
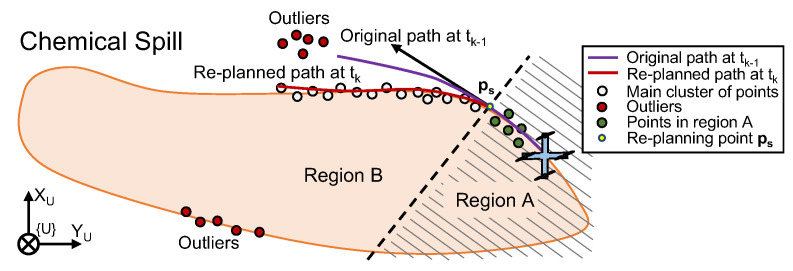
Pre-processing the point cloud and re-planning schematic.

Before using the 2-D point cloud to generate a path, it is important to pre-process the information provided in it. Consider, for instance, the example in Figure 6, where the quadrotor produces a 2-D point cloud, representing the boundary, at an arbitrary time-step $t_{k}$. In the point cloud, some points represent the chemical spill boundary in a region that is close to the vehicle—the region of interest, i.e., where the main cluster of points is expected to be located (in region B). The separation between regions A and B is defined by drawing a normal to the path at the point where the re-planning starts (defined formally in Section 6.3.1). Some points are outliers as a result of either noisy measurements or regions of the boundary that are not entirely captured by the field of view of the camera. The latter can be seen as disconnected from the main cluster and should be disregarded in the path planning process. According to Figure 6, the original path (in purple) obtained at time $t_{k-1}$ should be re-planned in order to obtain a new one (in red) that better fits the main cluster of points.

Unlike conventional motion planning problems, the main cluster of points does not have an explicit ordering, yielding a sequence of waypoints that the vehicle should visit sequentially in time—this information must be inferred. On the other hand, it is possible to define explicitly where the path re-planning process starts—at a point $p_{s}:=C\left(\gamma_{s}\right)$ arbitrarily further ahead of the drone’s position on the current curve C(γ), such that $\gamma_{drone}\leq \gamma_{s}$. Motivated by this example, and inspired by the work of Liu Y. et al. [27], the following pre-processing steps are introduced:

- Remove unused points that are behind the point $p_{s}$, i.e., points in region A;
- Order the remaining set of points and remove outliers in region B.

#### 6.2.1. Removing Unused Points

Consider $p_{s}\in R^{2}$ to be the point at which the path re-planning starts. In order to remove the points that are in region A, consider that $\psi_{s}$ is the tangent angle to the current path at $p_{s}$. A coordinate transformation can be applied to the 2-D point cloud $X:={\left\{X_{l}\right\}}_{l=1}^{L}\in R^{2}$, such that in a new reference frame, points that are behind $p_{s}$ (in region A) have a negative X-coordinate. This coordinate transformation is given by:

(53) $$X_{l}^{\circ }=R\left(\psi_{s}\right)\cdot \left(X_{l}-p_{s}\right),\forall l=1,...,L,$$

where $X_{l}^{\circ }={\left[X_{l}^{\circ x},X_{l}^{\circ y}\right]}^{T}$. Each point $X_{l}$ is discarded if $X_{l}^{\circ x}<0$. The points that belong to set X and are not discarded, and should be saved in a new set $X^{★}:={\left\{X_{j}\right\}}_{j=1}^{J}\in R^{2}$ with J≤L. The pseudo-code is shown in Algorithm 2.
**Algorithm 2** Remove points “behind” the re-planning point.1:Obtain a new 2-D point cloud $X:={\left\{X_{l}\right\}}_{l=1}^{L}\in R^{2}$;2:Define $p_{s}$ as the desired initial point for the re-planning to start;3:Define $\psi_{s}$ as the tangent angle to the current path at $t_{k}$ at $p_{s}$;4:Follow the procedure:5:**procedure**Remove unused points(X, $p_{s}$, $\psi_{s}$)6:    **for** l=1,...,L **do**7:        Compute $X_{l}^{\circ }$ according to (53);8:        **if** $X_{l}^{\circ x}<0$ **then**9:           Discard $X_{l}$;10:    **return** the new set $X^{★}:={\left\{X_{j}\right\}}_{j=1}^{J}\in R^{2}$ with J≤L.

#### 6.2.2. Ordering a Set of Points and Removing Outliers

**Figure 7:**
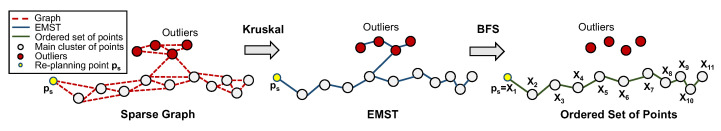
From sparse graph to an ordered list of points (example).

In order to avoid clustering outliers, reduce the point cloud to a curve-like shape, and extract some implicit ordering from the data, Lee I. [28] proposes an algorithm that seeks to extract a structure “as simple as possible” from the data, by resorting to an Euclidean Minimum Spanning Tree (EMST). Consider the unordered set of points $X^{★}$ obtained previously and a graph G=(V,E), such that $V=\{X_{j}=\left(x_{j},y_{j}\right)|j=1,...,J\}$ and $E=\{\left(X_{i},X_{j}\right)|i,j=1,...,J,i\neq j\}$. The EMST is a tree that connects all points in G with the weight of its edges corresponding to the Euclidean distance between each pair of points, that can be computed according to the very popular Kruskal’s algorithm. In order to reduce the time complexity of this process, a threshold distance $N_{J}$ can be used to define whether each pair of points is connected and a KDTree [29] can be used to compute a sparse graph G where each point has a limited set of neighbours, as shown in Figure 7.

To remove outliers and define a coarse path to follow, Breadth First Search (BFS) can be applied to the EMST, starting from $p_{s}$. This removal of outliers from the point cloud is crucial to avoid smaller clusters of points being considered later in the curve fitting problem. The resulting ordered list of points that forms the path with the highest number of points should be saved in a new ordered set $X^{†}:={\left\{X_{k}\right\}}_{k=1}^{K}\in R^{2}$. The proposed steps are summarised in Algorithm 3.
**Algorithm 3** Order a set of 2-D points.1:Add the desired initial point for the path $p_{s}$ to $X^{★}$;2:Define a threshold distance for the neighbours $N_{J}$;3:Follow the procedure:4:**procedure**Order Points($X^{★}$, $N_{J}$)5:    Construct a KDTree from $X^{★}$ and use $N_{J}$ as a distance threshold;6:    Create a graph G with *J* vertices and no edges;7:    **for** $X_{j},\;j=1,...,J$ **do**8:        Query the KDTree for the neighbours of $X_{j}$ and their euclidean distances;9:        Add the corresponding edges to the graph G;10:    Compute the MST of the graph G starting from vertex corresponding to $p_{s}$;11:    Compute the path with the highest number of points, starting at $p_{s}$ using BFS;12:    **return** the new ordered set of points $X^{†}:={\left\{X_{k}\right\}}_{k=1}^{K}\in R^{2}$.

### 6.3. Path Generation—Approximating the Point Cloud with a Parametric Curve

In order to have a suitable representation of a path that the proposed controllers can follow, it is a requirement to generate a curve that is smooth and at least $C^{2}$. In order to fulfil this requirement, the ordered set of points produced previously can be approximated by non-clamped uniform cubic B-splines, composed of multiple spline segments, where each segment is paramaterised by γ∈[0,1).

#### 6.3.1. Define the Number of Segments to Use

**Figure 8:**
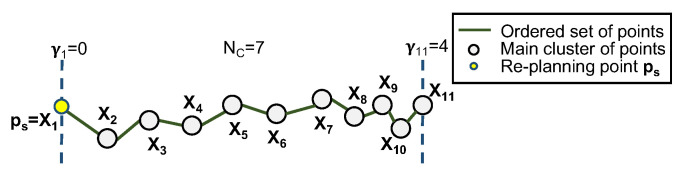
Ordered set of points with parametric values associated to them (example).

Consider now the ordered sequence of *K* points obtained via the application of Algorithms 2 and 3 to the original 2-D point cloud. In order to fit the points with a parametric curve, we are required to attribute to each point $X_{k}\in R^{2}$ a corresponding $\gamma_{k}$ in the target parametric curve. This problem could be formulated as a nonlinear optimisation problem—which is computationally demanding to solve for real-time applications. A non-optimal, but more efficient solution, proposed by Liu M. et al. [30] for Simultaneous Localisation and Mapping (SLAM) applications, is to consider $D_{X}$ to be the total distance between the points, given by:

(54) $$D_{X}:=\sum_{k=2}^{K}∥X_{k}-X_{k-1}∥,$$

and the corresponding vector of parametric values $\gamma ={\left[\gamma_{1},...,\gamma_{k}\right]}^{T}$ to be given by:

(55) $$\left\{\begin{array}{c} \gamma_{1}=0,\\ \gamma_{k}=\gamma_{k-1}+\frac{∥X_{k}-X_{k-1}∥}{D_{X}}\gamma_{max},k=2,...,K, \end{array}\right.$$

where $\gamma_{max}$ is the maximum parameter value of the parametric curve. For cubic B-splines, this number depends directly on the number of control points $N_{C}$ that the target curve will have, such that $\gamma_{max}=N_{C}-3$. The number of control points also dictates how many spline segments are used for the fitting problem. The optimal number of control points can be obtained by solving yet another nonlinear optimisation problem, but due to the real-time nature of the problem, this option is disregarded. Given that a uniform cubic B-spline must have at least four control points to define one segment, and that a low number of sections can under-fit a long set of points whilst a high number leads to over-fitting issues, this number should not be a static constant either. A non-optimal yet dynamic way of defining the number of control points $N_{C}$ is by taking:

(56) $$N_{C}:=max\left\{\left\lfloor \frac{D_{X}}{\rho }\right\rceil ,4\right\},$$

with (1/ρ)>0 being a control point’s density (tunning parameter defined a priori). A smaller ρ leads to a higher $N_{C}$. Applying this method to the previous example, and considering $N_{C}=7$, $\gamma_{max}=\gamma_{11}=4$, the result in Figure 8 is obtained.

#### 6.3.2. Fitting the Points with a Uniform Cubic B-Spline

For fitting the ordered set of points $X^{†}$ with a non-clamped uniform cubic B-spline C(γ,P), an optimisation problem is formulated. Consider the objective function given by:

(57) $$f\left(P\right):=\underset{goal}{\underset{︸}{\sum_{k=1}^{K}{∥C\left(\gamma_{k},P\right)-X_{k}∥}^{2}}}+F_{r},$$

with:

(58) $$F_{r}=\underset{\mathrm{regularisation}\;\mathrm{term}}{\underset{︸}{\lambda \int_{0}^{\gamma_{max}}{∥\frac{\partial C(\gamma ,P)}{\partial \gamma }∥}^{2}d\gamma +\beta \int_{0}^{\gamma_{max}}{∥\frac{\partial^{2}C\left(\gamma ,P\right)}{\partial \gamma^{2}}∥}^{2}d\gamma }},$$

where $P={\left[P_{0}^{x},...,P_{N_{c}-1}^{x},P_{0}^{y},...,P_{N_{c}-1}^{y}\right]}^{T}\in R^{2N_{c}}$ is the vector of control points that defines the target curve. The first term minimises the distance between the target B-spline curve and the set of points, whilst $F_{r}$ is a regularisation term and α,γ≥0 are the regularisation variables. The integral of the $L_{2}^{2}$ norm of the first derivative penalises the total length of the curve, while the integral of the $L_{2}^{2}$ norm of the second derivative penalises bends in the path. This objective function can also be expressed using vector notation, according to:

(59) $$f\left(P\right)=\underset{goal}{\underset{︸}{{∥B(\gamma )P-X∥}^{2}}}+\underset{\mathrm{regularisation}\;\mathrm{term}}{\underset{︸}{\lambda P^{T}R_{1}P+\beta P^{T}R_{2}P}},$$

where $X=[X_{1}^{x},...,X_{K}^{x},X_{1}^{y},...,X_{K}^{y}]$ denotes the points to fit, and $R_{1}$, $R_{2}$ are constant matrices that can be computed numerically (see Appendix D).

In order to define the new path, it would not suffice to discard the previously planned curve defined after $\gamma_{s}$ and minimise the objective function with respect to the control points. To guarantee $C^{2}$ continuity between the previous path and the newly planned one, linear equality constraints should be imposed on the values of $C^{new}\left(0\right)$, $C^{new^{\prime }}\left(0\right)$, and $C^{new^{\prime \prime }}\left(0\right)$ of the new curve. Moreover, it is a requirement to save the old curve up to $\gamma_{s}$, as it may still be in use by other vehicles in the network.

Consider the re-planning point $p_{s}$ introduced previously, chosen such that it corresponds to the transition between the spline segment that the virtual target of the drone is “sitting on”, and the next segment, according to:

(60) $$p_{s}=C^{old}\left(\gamma_{s}\right)\;\mathrm{with}\;\gamma_{s}=\left\lceil \gamma_{drone}\right\rceil ,$$

where $\gamma_{drone}$ corresponds to the quadrotor’s virtual target at time instant $t_{k}$. With this choice of $\gamma_{s}$, it is possible to take advantage of the local support property of B-splines and simplify the equality constraints of the problem, while at the same time simplifying the storage of the curves in memory.

Considering that $p_{s}$ is dictated by (60), the old curve segments that are described by parametric values such as $\gamma \geq \gamma_{s}$ should be discarded and replaced by a newer curve. Since each curve segment is defined by only four control points, discarding those segments is equivalent to removing control points with indexes $i\geq \gamma_{s}+3$ from the old control points vector. This operation results in a vector given by:

(61) $$P^{old}={\left[P_{0}^{x},P_{1}^{x},...,P_{\gamma_{s}}^{x},P_{\gamma_{s}+1}^{x},P_{\gamma_{s}+2}^{x},P_{0}^{y},P_{1}^{y},...,P_{\gamma_{s}}^{y},P_{\gamma_{s}+1}^{y},P_{\gamma_{s}+2}^{y}\right]}^{T}.$$

**Figure 9:**
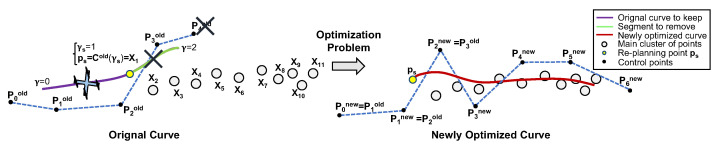
Solving the optimisation problem (example).

For the particular example in Figure 9, spline 1 (in green) should be discarded given that $\gamma_{drone}\in \left[0,1\right)$; hence, $\gamma_{s}=1$ and spline 0 are kept. To achieve this, all the control points with indexes i≥1+3 should be removed from the control points vector $P^{old}$, i.e., $P_{4}=\left(P_{4}^{x},P_{4}^{y}\right)$.

Making use of the local support property once more, it is known that $C^{2}$ continuity between two consecutive cubic spline segments is guaranteed, as long as the last three control points of the first segment coincide with the first three control points of the second segment. A trivial way of generating a new B-spline with guarantees of $C^{2}$ continuity in the transition with the old curve, without explicitly defining equality constraints on the derivatives of the function, is to solve the following optimisation problem:

(62) $$\begin{array}{cc} P^{new}\; & =\underset{P^{new}}{\mathrm{argmin}\;}f\left(P^{new}\right)\\ \mathrm{subject}\;\mathrm{to}\\ \; & \left[\begin{array}{c} P_{0}^{x\;new}\\ P_{1}^{x\;new}\\ P_{2}^{x\;new}\\ P_{0}^{y\;new}\\ P_{1}^{y\;new}\\ P_{2}^{y\;new} \end{array}\right]=\left[\begin{array}{c} P_{\gamma_{s}}^{x}\\ P_{\gamma_{s}+1}^{x}\\ P_{\gamma_{s}+2}^{x}\\ P_{\gamma_{s}}^{y}\\ P_{\gamma_{s}+1}^{y}\\ P_{\gamma_{s}+2}^{y} \end{array}\right], \end{array}$$

where $P^{new}={\left[P_{0}^{x\;new},...,P_{N_{C}-1}^{x\;new},P_{0}^{y\;new},...,P_{N_{C}-1}^{y\;new}\right]}^{T}$ is a new control points vector.

**Figure 10:**
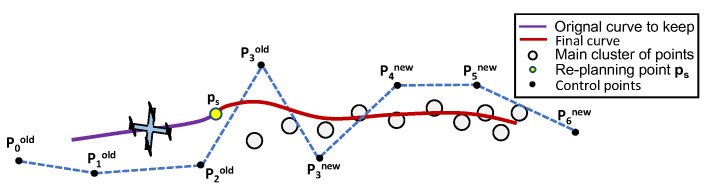
Final curve with control points concatenated (example).

To keep track of old and new curves, it is possible to concatenate only the new control points vector $P^{new}$ with the old control points vector $P^{old}$, ignoring the first three control points, i.e., $P_{0}^{new}$, $P_{1}^{new}$, and $P_{2}^{new}$, which are repeated as a result of the equality constraints imposed by (62). Applying this methodology to the previous example, the final control points vector is given according to Figure 10.

These series of procedures are summarised in Algorithm 4. For the sake of simplicity, the separation between the X- and Y-coordinates of the control points was omitted.
**Algorithm 4** Fitting the points—growing a uniform cubic B-spline1:Compute $D_{X}$, γ and $N_{C}$ according to Equations (54), (55), and (56), respectively;2:Consider $\gamma_{s}=\left\lceil \gamma_{t_{k}}\right\rceil$ and the original control points vector:
(63)$$P={\left[\begin{array}{c} P_{0},P_{1},...,P_{\gamma_{s}},P_{\gamma_{s}+1},P_{\gamma_{s}+2},P_{\gamma_{s}+3},P_{\gamma_{s}+4}...,P_{n} \end{array}\right]}^{T};$$3:Remove control points (corresponding to splines to be re-planned) from the original control points vector, such that:
(64)$$P^{old}={\left[\begin{array}{c} P_{0},P_{1},...,P_{\gamma_{s}},P_{\gamma_{s}+1},P_{\gamma_{s}+2} \end{array}\right]}^{T};$$4:Solve the optimisation problem in (62) and obtain a new vector with $N_{C}$ control points:
(65)$$\begin{array}{cc} & P^{new}={\left[\begin{array}{c} P_{0}^{new},P_{1}^{new},P_{2}^{new},...,P_{N_{C}-1}^{new} \end{array}\right]}^{T},\\ \mathrm{with} & P_{0}^{new}=P_{\gamma_{s}},P_{1}^{new}=P_{\gamma_{s}+1},P_{2}^{new}=P_{\gamma_{s}+2}; \end{array}$$5:Concatenate the new vector with the old vector (ignoring the first three control points, which are repeated):
(66)$$P^{final}={\left[\begin{array}{c} P_{0},P_{1},...,P_{\gamma_{s}},P_{\gamma_{s}+1},P_{\gamma_{s}+2},P_{3}^{new},...,P_{N_{C}-1}^{new} \end{array}\right]}^{T}.$$

### 6.4. From 2-D Path to Vehicle Formation

**Figure 11:**
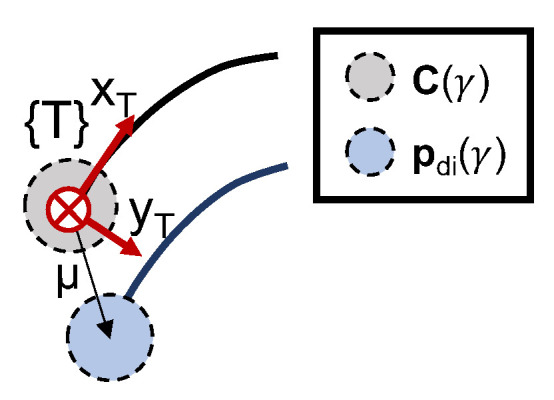
Formation vector.

To generate individual paths for each vehicle to follow, we can consider a reference path (obtained via the application of the previous algorithms) and offset each point according to an expression that captures a desired vehicle formation. Start by considering a formation vector denominated $\mu_{i}\in R^{3}$ for each vehicle *i*, with each distance defined in the tangential reference frame {T} to the virtual target’s position in the original curve, according to Figure 11. According to Xie et al. [31], it is possible to define a desired path for each vehicle given by:

(67) $$p_{di}\left(\gamma_{i}\right)=C\left(\gamma_{i}\right)+{}_{T}^{U}R\left(\gamma_{i}\right)\mu_{i},$$

where $p_{di}$ is the desired path for the vehicle *i*, $C(\gamma_{i})$ is the planned curve, and ${}_{T}^{U}R\left(\gamma_{i}\right)$ is a rotation matrix computed according to:

(68) $${}_{T}^{U}R\left(\gamma_{i}\right)=\left[r_{1}\left(\gamma_{i}\right),r_{3}\left(\gamma_{i}\right)\times r_{1}\left(\gamma_{i}\right),r_{3}\left(\gamma_{i}\right)\right],$$

with:

(69) $$\begin{array}{cccc} r_{1}\left(\gamma_{i}\right) & =\frac{\partial p_{d}/\partial \gamma }{∥\partial p_{d}/\partial \gamma ∥},\;\mathrm{with}\;∥\partial p_{d}/\partial \gamma ∥\neq 0 & r_{3}\left(\gamma_{i}\right) & =\frac{r_{d}-\left(r_{d}\cdot r_{1}\left(\gamma_{i}\right)\right)r_{1}\left(\gamma_{i}\right)}{∥r_{d}-\left(r_{d}\cdot r_{1}\left(\gamma_{i}\right)\right)r_{1}\left(\gamma_{i}\right)∥}, \end{array}$$

such that $r_{1}$ is the tangent to the curve. Moreover, since all vehicles will only be required to operate in a 2-D plane, a trivial definition for one of the axis of the tangential frame {T} is $r_{d}={\left[0,0,1\right]}^{T}$.

A path might not be not parameterised according to the arc length and, for B-splines in particular, each spline segment is such that γ∈[0,1]. Therefore, it is commonplace to define a constant required speed $V\leq V_{max}$ for the vehicle and let the desired speed profile for the virtual targets be given by:

(70) $$v_{L}\left(\gamma \right)=\frac{V}{∥p_{d}^{\prime }\left(\gamma \right)∥}.$$

## 7. Implementation Details

**Figure 12:**
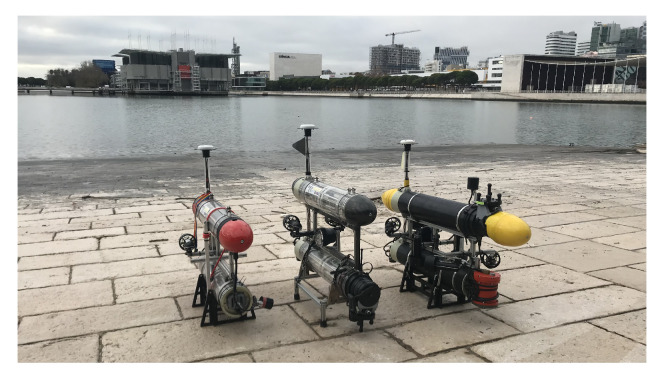
Real Medusa vehicles at Doca dos Olivais, Lisbon (Portugal).

To evaluate the performance of the proposed PF and CPF algorithms applied to marine ASVs, real water trials were conducted at Doca dos Olivais (Lisbon, Portugal) using the Medusa class of underactuated marine vehicles [14], shown in Figure 12. The vehicles used in the real trials were equipped with a GPS Astech MB100, a NavQuest600 Micro DVL, and a Vectornav VN-100T Attitude and Heading Reference System (AHRS). The operating system used during development was Ubuntu 18.04LTS along with ROS Melodic.

**Figure 13:**
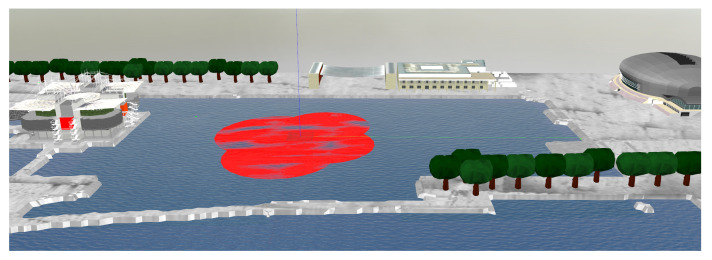
Simulated world of Doca dos Olivais with red chemical spill in Gazebo.

To analyse the performance of the proposed online path planning algorithm, a realistic simulation environment that closely resembles the Doca dos Olivais site was developed and incorporated into the Gazebo simulator. Given the main goal of having a fleet of vehicles encircling a chemical spill, is was necessary to overlay a red stain mesh on top of the ocean’s surface (Figure 13).

**Figure 14:**
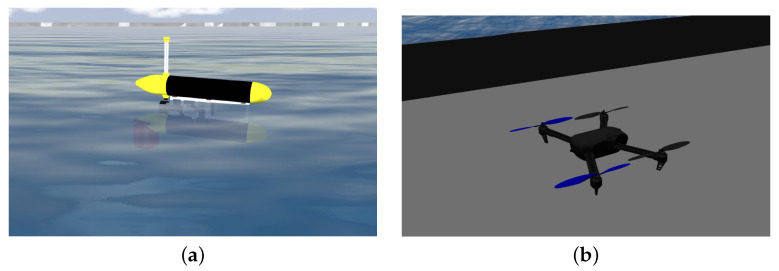
Simulated vehicles in gazebo: (**a**) Medusa ASV. (**b**) Iris quadrotor with a fixed camera.

For simulating the Medusa ASVs, a CAD model of the vehicles was incorporated into the simulator (Figure 14a). The virtual vehicle was also equipped with DVL, AHRS, and GPS sensors provided by the UUVSimulator plugin [16]. To simulate the quadrotor, the Iris vehicle provided by the PX4 SITL Gazebo plugin [15] was used; see Figure 14b.

**Figure 15:**
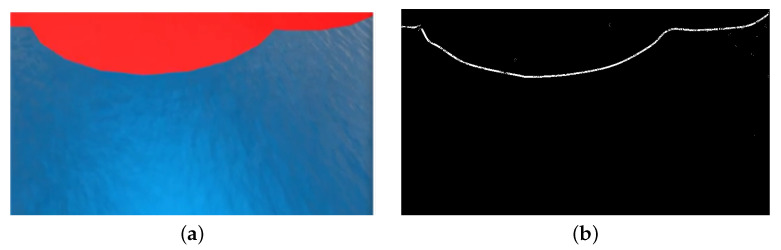
Simulated camera feed: (**a**) Quadrotor’s camera output. (**b**) Binary image.

The simulated quadrotor was equipped with a virtual camera mounted 21 mm below the vehicle’s center of mass and with a pitch angle of $-45^{\circ }$, pointing downwards, and produced an image with a resolution of 640×480 px, according to Figure 15a. Its intrinsic parameters are given by:

(71) $$\left\{\begin{array}{c} \left(c_{x},c_{y}\right)=\left(320.5,240.5\right)\\ \left(f_{d}s_{x},f_{d}s_{y}\right)=\left(381.4,381.4\right). \end{array}\right.$$

Given assumption 5, the detection of the boundary region between the spill and the ocean surface was out of the scope of this work. Therefore, we resorted to OpenCV library [32] to mask and threshold the red colours in the image feed. After this step, the Canny edge detection algorithm was applied to the binary image to retrieve the pixels corresponding to the boundary, according to Figure 15b. To solve the optimisation problem proposed in Section 6.3.2, we resorted to Scipy’s SQP solver [33].

**Figure 16:**
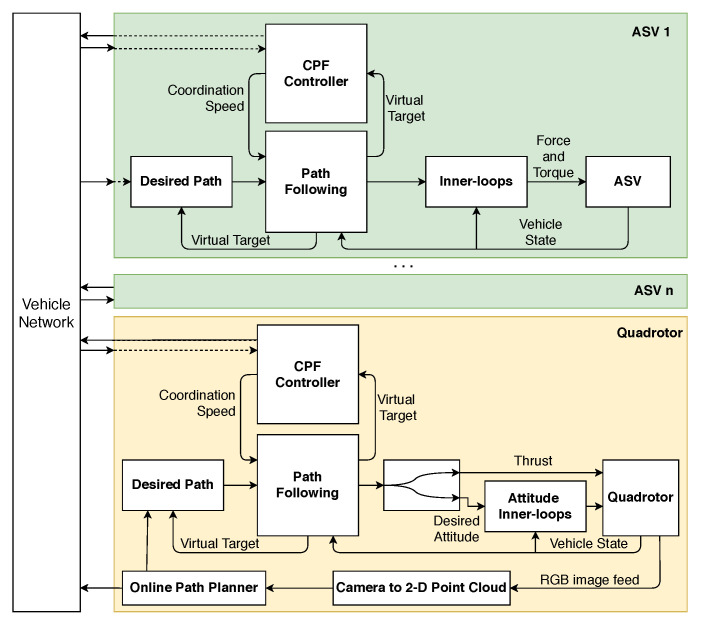
Planning and control architecture.

The entire system architecture is shown in Figure 16. The inner-loop controls adopted for the quadrotor were the ones already provided by PX4, while for the ASV, we resorted to PID inner-loop controllers to steer the vehicles.

## 8. Experimental and Simulation Results

In this section, we present some real experimental results regarding the PF and CPF controllers applied to two Medusa ASV vehicles. In addition, realistic 3-D simulation results are also presented for the case study where two Medusa vehicles were required to perform a CPF mission on a pre-defined path with a quadrotor, in a leader–follower formation. Finally, a third case study is presented, where a simulated quadrotor had to detect the boundary of a chemical spill, and plan, in real-time, a path for both itself and a Medusa ASV to follow cooperatively. The control gains adopted are available in Appendix E.

### 8.1. Cpf with ETC between 2 Medusa Vehicles (Real)

**Figure 17:**
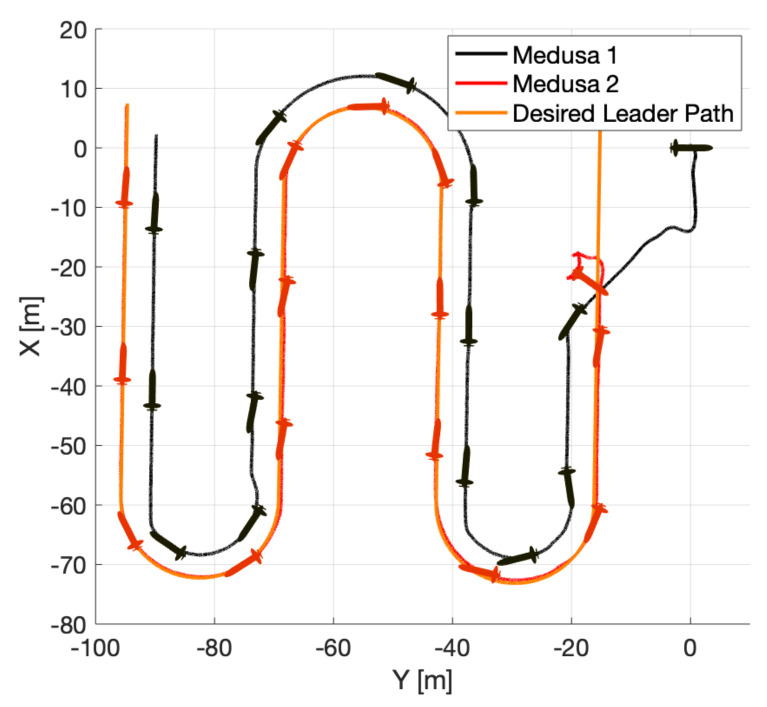
Real CPF mission with 2 Medusa vehicles.

For the real trial, performed at Doca dos Olivais (Lisbon, Portugal), two Medusa vehicles were required to perform a lawn-mowing mission cooperatively at the surface of the water, according to Figure 17. The black vehicle (Medusa 1) was required to follow the leader (Medusa 2) according to the formation vector $\mu ={\left[-5,-5,0\right]}^{T}$. Both vehicles were required to follow the path at V=0.5 m/s and communications were bi-directional.

**Figure 18:**
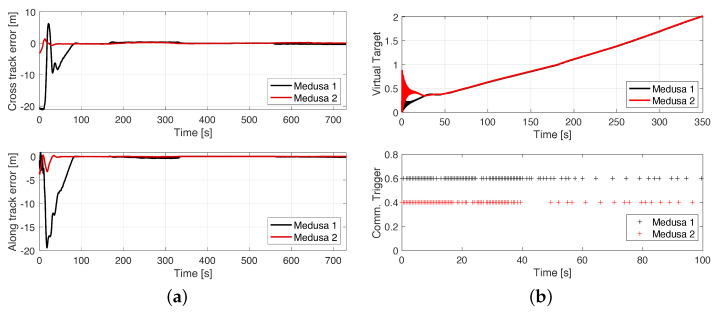
CPF with 2 real Medusa vehicles: (**a**) X–Y view. (**b**) Communication metrics.

According to the results in Figure 18a, the along-track error of Medusa 1 increases quickly as the virtual target tries not only to minimise the distance to the vehicle but also its distance to its neighbour’s (Medusa 2) virtual target. As the vehicles start to move, this error starts to decrease, and according to Figure 18b, after approximately 50 s, the vehicles align themselves according to the desired formation, approach the desired speed profile and, as a consequence, the rate of information exchange decreases. This decrease in the rate of communication is due to the bank of estimators for the virtual targets running in each vehicle being able to better predict the evolution of the virtual targets.

### 8.2. Cpf with ETC between a Quadrotor and Medusa Vehicles (Simulation)

**Figure 19:**
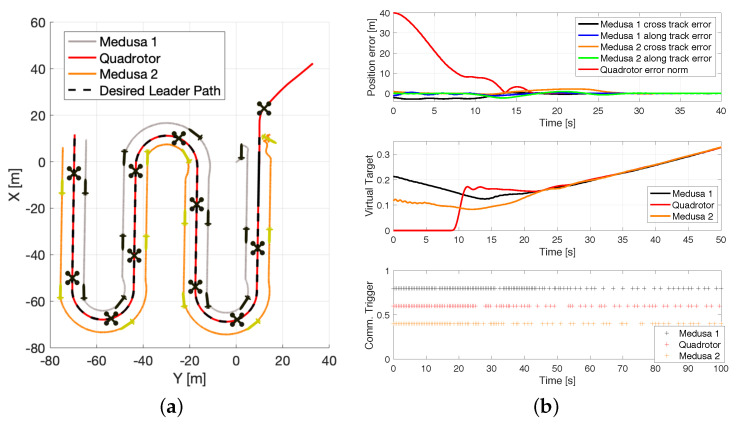
CPF with simulated Iris and Medusa vehicles: (**a**) X–Y view. (**b**). Performance metrics.

For this case study, a CPF mission was performed such that a simulated quadrotor and two Medusa vehicles were required to perform a lawn-mowing mission, according to Figure 19a. In this experiment, the aircraft was required to fly at a fixed altitude of 30 m; the formation vector for Medusa 1 was given by $\mu_{1}={\left[-5,5,0\right]}^{T}$ m, and for Medusa 2, by $\mu_{2}={\left[-5,-5,0\right]}^{T}$ m, leading to a triangular formation with 2 ASVs side by side, behind the quadrotor. In this experiment, there was bi-directional communication between the pairs of vehicles: (quadrotor, Medusa 1) and (quadrotor, Medusa 2). From the results in Figure 19b, it is observable that the vehicles converge to their desired formation at around 25 s. After this period of time, the position error converges to a neighbourhood of zero and the virtual target speeds converge to their desired value. As a consequence, the number of communication events between the vehicles drops as the bank of observers in each vehicle can more accurately track the state of the virtual target of their peers.

### 8.3. Boundary Encircling with a Quadrotor and a Medusa Vehicle (Simulation)

**Figure 20:**
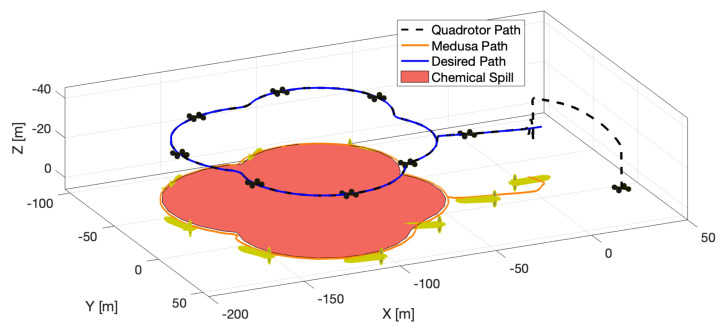
3-D view of simulated boundary encircling mission with Iris and Medusa vehicles.

For the last simulated experiment, the quadrotor was required to start the same lawn-mowing that was adopted for the mission with one Medusa ASV. As soon as a chemical spill boundary was detected in the drone’s image stream, the quadrotor was required to start the path planning algorithm at a rate of 1 Hz and send the most up-to-date path to the ASV, according to Figure 20. The drone was required to follow the path at 30 m of altitude with a desired constant speed of 0.5 m/s. Since the quadrotor was equipped with a fixed-mounted camera, it was also required to align its yaw angle with the tangent to the path in order not to lose sight of the boundary being followed.

**Figure 21:**
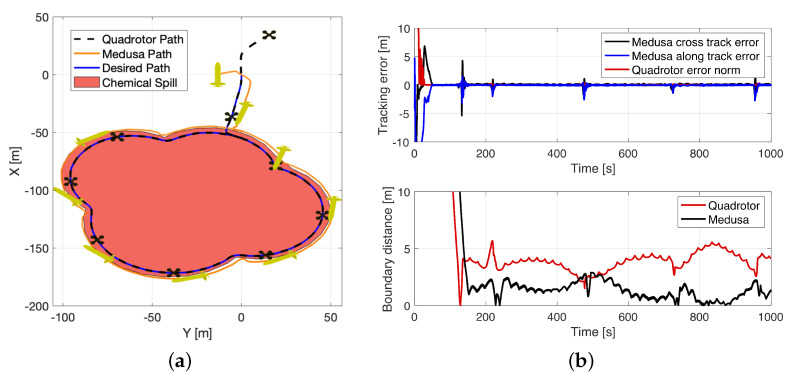
Boundary encircling with simulated Iris and Medusa vehicles: (**a**) X–Y view. (**b**) Performance metrics.

In order to guarantee that the path further ahead could be generated for the ASV to follow, it was desirable for the marine vehicle to follow the the quadrotor from behind, i.e., with a formation vector $\mu ={\left[-5,5,0\right]}^{T}$ m. In Figure 21a, a top-down view of the executed mission is shown. In Figure 21b, plots of the PF errors are provided along with the norm of the horizontal distance of each vehicle to the real boundary being followed. It is observable that the tracking error only increased in zones where the chemical spill had a crease. This is justified by the fact that the Medusa vehicle, when performing tight turns, was not able to cope with its virtual target speed and slowed down, leading to sudden spikes in the along-track error. These tracking errors were instantly compensated by the adaptive virtual target dynamics, which attempted to minimise the distance between itself and the vehicle. It is also observable that the norm of the distance between the marine vehicle and the chemical spill is much lower than its aerial counterpart, with the Medusa always following the boundary from its outskirts, due to the formation vector adopted.

From Figure 21b, it is also evident that the horizontal distance between the real drone’s position and the boundary is bounded by 6m. This result is to be expected, as the altitude estimates are mainly provided by the simulated GPS system and small errors in the estimated attitude, especially yaw angle, will lead to errors of several meters in the generated 2-D point cloud. Due to the type of application at hand, and given that it is typical to have errors of several meters in underwater scenarios, these errors are considered within an acceptable range. In addition, the small oscillations in the boundary distance plot result from the simulated chemical spill boundary mesh being a composition of discrete lines which are picked up by the drone’s camera.

**Figure 22:**
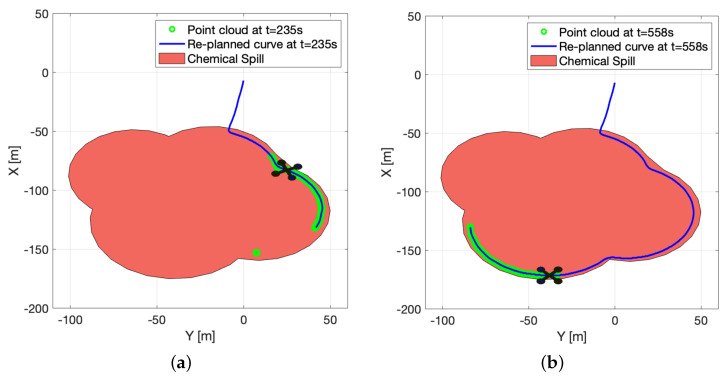
Path generation (**a**) Time = 235 s. (**b**) Time = 558 s.

In Figure 22, a plot of the point cloud generated by the algorithm is shown at two different time instants (in green), as well as the corresponding planned B-spline paths (in blue). Note that in Figure 22a, some of the green dots further away from the vehicle were discarded by the planning algorithm, as they were too far away from the main cluster of points.

## 9. Conclusions and Future Work

This paper addressed the problem of encircling an environmental boundary caused by a chemical spill using a team of robots composed of an aerial quadrotor and Medusa marine vehicles. The path following problem was introduced, and a non-linear control law derived for the ASV, exploiting the technique described in P. Aguiar and F. Vanni [10,11,12]. Inspired by this control law, a new one was derived for a quadrotor following the same methodology, with some key differences due to the nature of the aircraft. For the section that followed, the CPF problem was formulated and a proposal to solve the problem was presented, such that the synchronisation controller was distributed and the same for all vehicles (aerial and marine) using event-triggered communications based on previous work by N. Hung and F. Rego [13]. In addition, a new real-time path planning algorithm was developed that made use of the camera sensor onboard of the quadrotor to have a local view of the boundary and generate a point cloud expressed in the inertial frame. This data was then used to solve an optimisation problem which generates a B-spline-based path that grows dynamically as the drone moves along the boundary and acquires more data. The path is then shared with all ASV vehicles in the network in real time. The proposed algorithms were implemented in ROS, and a 3-D virtual scenario was generated, allowing for a mixture of real and simulated results. Future work includes making the height at which the quadrotor operates dynamic and introducing curvature limits as inequality constraints to the path planning problem, as well as obstacle avoidance before carrying out integrated experiments with real vehicles.

## Data Availability

The path planning library that implements the algorithms proposed in Section 6 is available at https://github.com/MarceloJacinto/BSplineFit (accessed on 8 March 2022).

## Appendix A. Proof of Preposition 1

**Proof.** Consider the candidate Lyapunov Function given by:

(A1) $$V_{1}\left(e_{p}\right)=\frac{1}{2}{\left(e_{p}-\delta \right)}^{T}\left(e_{p}-\delta \right).$$

Taking the first time derivative of (A1) and replacing in (17) and (14) leads to:

(A2) $$\begin{array}{cc} {\dot{V}}_{1}\left(e_{p}\right)={\left(e_{p}-\delta \right)}^{T}( & -S\left(r\right)\left(e_{p}-\delta \right)+\Delta u+\left[\begin{array}{c} 0\\ v \end{array}\right]\\ + & v_{c}-{}_{U}^{B}R\left(\psi \right)\frac{\partial p_{d}\left(\gamma \right)}{\partial \gamma }\left(e_{\gamma }+v_{d}\left(\gamma ,t\right)\right)). \end{array}$$

Taking into account the properties of the skew-symmetric matrix S:

(A3) $${\left(e_{p}-\delta \right)}^{T}S\left(r\right)\left(e_{p}-\delta \right)=0.$$

Replacing (20) and (21) in ${\dot{V}}_{1}$ yields:

(A4) $$\begin{array}{cc} {\dot{V}}_{1}\left(e_{p}\right) & ={\left(e_{p}-\delta \right)}^{T}\left(\Delta \left(\tilde{u}+u_{d}\right)+\left[\begin{array}{c} 0\\ v \end{array}\right]+{\tilde{v}}_{c}+{\hat{v}}_{c}-{}_{U}^{B}R\left(\psi \right)\frac{\partial p_{d}\left(\gamma \right)}{\partial \gamma }\left(e_{\gamma }+v_{d}\left(\gamma ,t\right)\right)\right)\\ \; & =-{\left(e_{p}-\delta \right)}^{T}K_{p}\sigma \left(e_{p}-\delta \right)-{\left(e_{p}-\delta \right)}^{T}{}_{U}^{B}R\left(\psi \right)\frac{\partial p_{d}\left(\gamma \right)}{\partial \gamma }e_{\gamma }+\Delta \tilde{u}+{\tilde{v}}_{c}. \end{array}$$

By taking a backstepping approach, consider a second candidate Lyapunov function:

(A5) $$V_{2}\left(e_{p},e_{\gamma }\right)=V_{1}\left(e_{p}\right)+\frac{1}{2}e_{\gamma }^{2}.$$

Taking the first derivative and replacing in the control law (22) for the virtual target, we obtain:

(A6) $$\begin{array}{cc} {\dot{V}}_{2}\left(e_{p},e_{\gamma }\right)= & {\dot{V}}_{1}\left(e_{p}\right)+e_{\gamma }\left(\ddot{\gamma }-{\dot{v}}_{d}\left(\gamma ,t\right)\right)\\ = & -{\left(e_{p}-\delta \right)}^{T}K_{p}\sigma \left(e_{p}-\delta \right)-k_{\gamma }e_{\gamma }^{2}+\Delta \tilde{u}+{\tilde{v}}_{c}\\ \leq & -\left(1-\theta \right){\left(e_{p}-\delta \right)}^{T}K_{p}\sigma \left(e_{p}-\delta \right)-\theta {\left(e_{p}-\delta \right)}^{T}K_{p}\sigma \left(e_{p}-\delta \right)\\ \; & -k_{\gamma }{\left|e_{\gamma }\right|}^{2}+∥e_{p}-\delta ∥∥\Delta \tilde{u}+{\tilde{v}}_{c}∥, \end{array}$$

where 0<θ<1. The term:

(A7) $$\begin{array}{cc} \; & -\theta {\left(e_{p}-\delta \right)}^{T}K_{p}\sigma \left(e_{p}-\delta \right)+∥e_{p}-\delta ∥∥\Delta \tilde{u}+{\tilde{v}}_{c}∥\\ \; & =-\theta {\left(e_{p}-\delta \right)}^{T}K_{p}\frac{e_{p}-\delta }{∥e_{p}-\delta ∥}\sigma \left(∥e_{p}-\delta ∥\right)+∥e_{p}-\delta ∥∥\Delta \tilde{u}+{\tilde{v}}_{c}∥, \end{array}$$

will be ≤0 if:

(A8) $$\theta \lambda_{min}\left(K_{p}\right)\sigma \left(∥e_{p}-\delta ∥\right)\geq ∥\Delta \tilde{u}+{\tilde{v}}_{c}∥,$$

which, in turn, implies that:

(A9) $$∥e_{p}-\delta ∥\geq \sigma^{-1}\left(\frac{1}{\theta \lambda_{min}\left(K_{p}\right)}∥\Delta \tilde{u}+{\tilde{v}}_{c}∥\right),$$

and:

(A10) $${\dot{V}}_{2}\leq -\left(1-\theta \right){\left(e_{p}-\delta \right)}^{T}K_{p}\sigma \left(e_{p}-\delta \right)-k_{\gamma }{\left|e_{\gamma }\right|}^{2},$$

as the right side of inequality (A9) can be made arbitrarily small through the choice of the gain matrix $K_{p}$. It follows directly from H. Khalil ([34], Theorem 4.19) that the controlled system is ISS. □

## Appendix B. Proof of Preposition 2

**Proof.** Consider the candidate Lyapunov function given by:

(A11) $$V_{1}\left(e_{p}\right):=\frac{1}{2}e_{p}^{T}e_{p}.$$

Taking the first derivative of (A11) and replacing in (24)–(26), yields:

(A12) $${\dot{V}}_{1}\left(e_{p}\right)=-e_{p}^{T}K_{1}e_{p}+e_{p}^{T}\left(z-\frac{\partial p_{d}}{\partial \gamma }e_{\gamma }\right).$$

With a view to applying backstepping techniques, consider:

(A13) $$V_{2}\left(e_{p},e_{v}\right):=V_{1}\left(e_{p}\right)+\frac{1}{2}z^{T}z.$$

Replacing (26), (27), and (29) in ${\dot{V}}_{2}$ yields:

(A14) $$\begin{array}{cc} {\dot{V}}_{2}= & -e_{p}^{T}K_{1}e_{p}+e_{p}^{T}z-e_{p}^{T}\frac{\partial p_{d}}{\partial \gamma }e_{\gamma }\\ \; & +z^{T}\left(u+d-h\left(\gamma \right)\left(e_{\gamma }+v_{d}\left(\gamma ,t\right)\right)-\frac{\partial p_{d}}{\partial \gamma }{\dot{v}}^{coord}\left(t\right)+K_{1}e_{v}-K_{1}\frac{\partial p_{d}}{\partial \gamma }e_{\gamma }\right). \end{array}$$

By replacing (35)–(37) in the Lyapunov time derivative, it follows that:

(A15) $${\dot{V}}_{2}=-e_{p}^{T}K_{1}e_{p}-z^{T}K_{2}z-e_{p}^{T}\frac{\partial p_{d}}{\partial \gamma }e_{\gamma }-z^{T}\left(h\left(\gamma \right)+K_{1}\frac{\partial p_{d}}{\partial \gamma }\right)e_{\gamma }+z^{T}\left(\tilde{u}+\tilde{d}\right).$$

Consider a third candidate Lyapunov, obtained by backstepping, defined as:

(A16) $$V_{3}:=V_{2}+\frac{1}{2}e_{\gamma }^{2}.$$

Taking its derivative, with respect to time, and replacing in (38), we obtain:

(A17) $${\dot{V}}_{3}=-e_{p}^{T}K_{1}e_{p}-z^{T}K_{2}z-k_{\gamma }e_{\gamma }^{2}+z^{T}\left(\tilde{u}+\tilde{d}\right).$$

Consider one last backstepping that involves the construction of:

(A18) $$V_{4}=V_{3}+\frac{1}{2}{\tilde{d}}^{T}K_{d}^{-1}\tilde{d}.$$

Taking its time derivative, and taking into consideration (33):

(A19) $$\begin{array}{cc} {\dot{V}}_{4} & =-e_{p}^{T}K_{1}e_{p}-z^{T}K_{2}z-k_{\gamma }e_{\gamma }^{2}+\underset{\leq 0}{\underset{︸}{{\tilde{d}}^{T}\left(z-\mathrm{Proj}\left(z,\hat{d}\right)\right)}}+z^{T}\tilde{u}\\ \; & \leq -W\left(e_{p},e_{v},e_{\gamma }\right)+z^{T}\tilde{u}. \end{array}$$

Assuming that the quadrotor is equipped with a generic inner loop that is capable of keeping the tracking error $\tilde{u}$ small and bounded, the right side of inequality (A19) can be made small enough such that the controlled system is stable. A more in-depth stability analysis can be conducted for the inner–outer loop control system, but this will be dependent directly on the type of inner loop adopted. This results from the fact that the desired accelerations $u_{d}$ must be decoupled in a set of desired thrusts and attitudes for the quadrotor to track. In order to simplify the designed control law $u_{d}$, consider the final algebraic manipulation:

(A20) $$\begin{array}{cc} u_{d}^{⋄} & =-\hat{d}+h\left(\gamma \right)v_{d}\left(\gamma ,t\right)+\frac{\partial p_{d}}{\partial \gamma }{\dot{v}}^{coord}\left(t\right)-K_{1}e_{v}-e_{p}-K_{2}z\\ \; & =-\hat{d}+h\left(\gamma \right)v_{d}\left(\gamma ,t\right)+\frac{\partial p_{d}}{\partial \gamma }{\dot{v}}^{coord}\left(t\right)-e_{v}\underset{K_{v}}{\underset{︸}{\left(K_{1}+K_{2}\right)}}-e_{p}\underset{K_{p}}{\underset{︸}{\left(I+K_{1}K_{2}\right)}}. \end{array}$$

□

## Appendix C. Proof of Preposition 3

**Proof.** Consider that:

(A21) $$v_{L}\left(\gamma \right)=v_{L}1+{\tilde{v}}_{L},$$

where ${\tilde{v}}_{L}$ is a bounded and arbitrarily small term that accounts for a transient period in which the vehicles are on different sections of the path, with slightly different desired speed profiles. Replacing (39), the speed correction term proposed in (46), and (A21) in (42) yields:

(A22)

where d is a disturbance that results from combining the terms dependent on ${\tilde{v}}_{L}$ and $\tilde{\gamma }$. Consider the Laplacian matrix *L*, expressed in canonical Jordan form as:

(A23) $$L=V\Lambda V^{-1},$$

and the change of variables:

(A24) $$\bar{\varepsilon }=V^{-1}\varepsilon .$$

Applying (A24) to (A22) yields:

(A25) $$\dot{\bar{\varepsilon }}=-k_{\varepsilon }\Lambda \left(\bar{\varepsilon }+\bar{d}\right),\;\mathrm{with}\;\bar{d}=V^{-1}d.$$

It is possible to decompose the above equality according to the notation:

(A26) $$\left[\begin{array}{c} {\dot{\bar{\varepsilon }}}_{1}\\ {\dot{\bar{\varepsilon }}}_{2} \end{array}\right]=\left[\begin{array}{c} 0\\ -k_{\varepsilon }\Lambda_{2}\left({\bar{\varepsilon }}_{2}+{\bar{d}}_{2}\right) \end{array}\right],$$

where the first half of the vector denotes the term that depends on the null eigenvalue of the Laplacian, while the second term is a vector that depends only on the positive eigenvalues of the Laplacian. Consider now the candidate Lyapunov function:

(A27) $$V_{{\bar{\varepsilon }}_{2}}=\frac{1}{2}{\bar{\varepsilon }}_{2}^{T}{\bar{\varepsilon }}_{2},$$

and its time derivative, given by:

(A28) $$\begin{array}{cc} {\dot{V}}_{{\bar{\varepsilon }}_{2}} & =-k_{\varepsilon }{\bar{\varepsilon }}_{2}^{T}\Lambda_{2}\left({\bar{\varepsilon }}_{2}+{\bar{d}}_{2}\right)\\ \; & =-\left(1-\theta \right)k_{\varepsilon }{\bar{\varepsilon }}_{2}^{T}\Lambda_{2}{\bar{\varepsilon }}_{2}-\theta k_{\varepsilon }{\bar{\varepsilon }}_{2}^{T}\Lambda_{2}{\bar{\varepsilon }}_{2}-k_{\varepsilon }{\bar{\varepsilon }}_{2}^{T}\Lambda_{2}{\bar{d}}_{2}, \end{array}$$

where 0<θ<1. The term:

(A29) $$-\theta k_{\varepsilon }{\bar{\varepsilon }}_{2}^{T}\Lambda_{2}{\bar{\varepsilon }}_{2}-k_{\varepsilon }{\bar{\varepsilon }}_{2}^{T}\Lambda_{2}{\bar{d}}_{2},$$

will be ≤0 if:

(A30) $$∥{\bar{\varepsilon }}_{2}∥\geq \frac{1}{\theta }∥{\bar{d}}_{2}∥,$$

and, therefore:

(A31) $${\dot{V}}_{{\bar{\varepsilon }}_{2}}\leq -\left(1-\theta \right)k_{\varepsilon }{\bar{\varepsilon }}_{2}^{T}\Lambda_{2}{\bar{\varepsilon }}_{2}.$$

The term $∥\tilde{\gamma }∥$ can be made arbitrarily small by controlling the gains that dictate the broadcasting scheme. Moreover, the term ${\tilde{v}}_{L}$ can be dominated by a proper choice $k_{\varepsilon }$. Hence, $∥d∥$ can be made arbitrarily small and, thus, $∥{\bar{d}}_{2}∥$ can be as well. It follows directly from H. Khalil ([34], Theorem 4.19) that the controlled system is ISS with respect to the error vector ε and the inputs $\tilde{\gamma }$ and ${\tilde{v}}_{L}$. □

## Appendix D. Computing the Regularisation Term Using Vectorial Notation

Consider the simplest unclamped uniform cubic B-spline with only one segment, such that γ∈[0,1] and is described by (6). Then, its first derivative $C^{\prime }\left(\gamma \right)$ is given by:

(A32) $$\frac{\partial C^{x/y}}{\partial \gamma }\left(\gamma \right)=\underset{B^{\prime }\left(\gamma \right)}{\underset{︸}{\underset{T(\gamma )}{\underset{︸}{\left[\begin{array}{cccc} \gamma^{2} & \gamma & 1 & 0 \end{array}\right]}}\underset{M}{\underset{︸}{\frac{1}{6}\left[\begin{array}{cccc} 3 & 0 & 0 & 0\\ 0 & 2 & 0 & 0\\ 0 & 0 & 1 & 0\\ 0 & 0 & 0 & 0 \end{array}\right]\left[\begin{array}{cccc} -1 & 3 & -3 & 1\\ 3 & -6 & 3 & 0\\ -3 & 0 & 3 & 0\\ 1 & 4 & 1 & 0 \end{array}\right]}}}}\underset{P}{\underset{︸}{\left[\begin{array}{c} P_{0}^{x/y}\\ P_{1}^{x/y}\\ P_{2}^{x/y}\\ P_{3}^{x/y} \end{array}\right]}}.$$

Therefore, the term $\int_{\gamma }{∥C^{\prime }\left(\gamma \right)∥}^{2}d\gamma$ is computed according to:

(A33) $$\begin{array}{cc} \int_{\gamma }{∥C^{\prime }\left(\gamma \right)∥}^{2}d\gamma & =\int_{\gamma }{\left(B^{\prime }\left(\gamma \right)P\right)}^{T}\left(B^{\prime }\left(\gamma \right)P\right)d\gamma \\ \; & =\int_{0}^{1}P^{T}B^{\prime }{\left(\gamma \right)}^{T}B^{\prime }\left(\gamma \right)Pd\gamma \\ \; & =P^{T}M^{T}\left[\int_{0}^{1}T{\left(\gamma \right)}^{T}T\left(\gamma \right)d\gamma \right]MP. \end{array}$$

Further, note that:

(A34) $$\begin{array}{c} T{\left(\gamma \right)}^{T}T\left(\gamma \right)=\left[\begin{array}{cccc} \gamma^{4} & \gamma^{3} & \gamma^{2} & 0\\ \gamma^{3} & \gamma^{2} & \gamma & 0\\ \gamma^{2} & \gamma & 1 & 0\\ 0 & 0 & 0 & 0 \end{array}\right] \end{array}$$

and, as a consequence:

(A35) $$\int_{0}^{1}T{\left(\gamma \right)}^{T}T\left(\gamma \right)d\gamma =Q=\left[\begin{array}{cccc} 1/5 & 1/4 & 1/3 & 0\\ 1/4 & 1/3 & 1/2 & 0\\ 1/3 & 1/2 & 1 & 0\\ 0 & 0 & 0 & 0. \end{array}\right]$$

Hence, for the simplest case of a single B-spline segment, it is known that:

(A36) $$\int_{\gamma }{∥C^{\prime }\left(\gamma \right)∥}^{2}d\gamma =P^{T}\underset{R_{1}}{\underset{︸}{M^{T}QM}}P.$$

The easiest way to extend this technique to a B-spline with *n* segments is to consider the modified vector $T\left(\gamma \right)={\left[{\left(\gamma -i\right)}^{2},\left(\gamma -i\right),1,0\right]}^{T}$, where i=⌊γ⌋, according to the notation introduced in Section 2.3. Then, since (γ−i)∈[0,1], one can compute individually, for each segment, intermediate matrices $R_{1}^{i}$, according to (A36). Due to the locality property of B-splines, it is possible to “stack” these intermediate matrices to form the final matrix $R_{1}$. An analogous rationale can be applied to compute $R_{2}$.

## Appendix E. Controller Gains Adopted

The controller gains used to obtain the results in Section 8 are presented in Table A1.

**Table A1:** Controller and path planning gains.

Currents Observer (ASV)		Projection Operator (Quadrotor)	
$k_{1}$	2.0	$K_{d}$	diag(0.5,0.5,0.2)
$k_{2}$	0.2	ς and β	10.0
**Path Following (ASV)**		**Path Following (Quadrotor)**	
$K_{p}$	diag(0.5,0.5)	$K_{p}$	diag(5.5,5.5,5.5)
δ	−1.0	$K_{d}$	diag(4.5,4.5,4.0)
$k_{\gamma }$	0.5	$k_{\gamma }$	0.5
**Cooperative Path Following**		**Path Planning**	
$k_{\varepsilon }$	1.0	$N_{J}$	0.6 m
*c*	0.001	1/ρ	4.0
*b*	5.0	λ	0.05
α	1.0	β	0.01
